# Blood-based biomarkers of Alzheimer’s disease and incident dementia in the community

**DOI:** 10.1038/s41591-025-03605-x

**Published:** 2025-03-26

**Authors:** Giulia Grande, Martina Valletta, Debora Rizzuto, Xin Xia, Chengxuan Qiu, Nicola Orsini, Matilda Dale, Sarah Andersson, Claudia Fredolini, Bengt Winblad, Erika J. Laukka, Laura Fratiglioni, Davide L. Vetrano

**Affiliations:** 1 Aging Research Center, Department of Neurobiology, Care Sciences and Society, Karolinska Institutet and Stockholm University, Stockholm, Sweden; 2 Stockholm Gerontology Research Center, Stockholm, Sweden; 3 Division of Neurogeriatrics, Department of Neurobiology, Care Sciences and Society, Karolinska Institutet, Stockholm, Sweden; 4 Department of Global Public Health, Karolinska Institutet, Stockholm, Sweden; 5 Affinity Proteomics Stockholm, Science for Life Laboratory, Department of Protein Science, School of Engineering Sciences in Chemistry, Biotechnology and Health (CBH), Royal Institute of Technology (KTH), Solna, Sweden; 6 Theme Inflammation and Aging, Karolinska University Hospital, Huddinge, Sweden

**Keywords:** Predictive markers, Diagnostic markers, Alzheimer's disease

## Abstract

Evidence regarding the clinical validity of blood biomarkers of Alzheimer’s disease (AD) in the general population is limited. We estimated the hazard and predictive performance of six AD blood biomarkers for incident all-cause and AD dementia—the ratio of amyloid-β 42 to amyloid-β 40 and levels of tau phosphorylated at T217 (p-tau217), tau phosphorylated at T181 (p-tau181), total tau, neurofilament light chain (NfL), and glial fibrillary acidic protein (GFAP)—in a cohort of 2,148 dementia-free older adults from Sweden, who were followed for up to 16 years. In multi-adjusted Cox regression models, elevated baseline levels of p-tau181, p-tau217, NfL, and GFAP were associated with a significantly increased hazard for all-cause and AD dementia, displaying a non-linear dose–response relationship. Elevated concentrations of p-tau181, p-tau217, NfL, and GFAP demonstrated strong predictive performance (area under the curve ranging from 70.9% to 82.6%) for 10-year all-cause and AD dementia, with negative predictive values exceeding 90% but low positive predictive values (PPVs). Combining p-tau217 with NfL or GFAP further improved prediction, with PPVs reaching 43%. Our findings suggest that these biomarkers have the potential to rule out impending dementia in community settings, but they might need to be combined with other biological or clinical markers to be used as screening tools.

## Abstract (web summary)

In a community-based cohort of more than 2,000 older adults from Sweden, blood biomarkers for Alzheimer’s disease were able to predict dementia up to 16 years before disease onset.

## Main

Early detection of dementia, including Alzheimer's disease (AD) dementia, is essential for timely intervention and for improving patient care and outcomes^1^. Blood-based biomarkers have recently emerged as promising predictive tools for dementia. Compared with other ways of measuring AD biomarkers (for example, cerebrospinal fluid (CSF) collection or brain positron emission tomography (PET) imaging), blood sampling is less invasive, scalable and largely accessible in community settings, leading to time and cost savings^2,3^.

Blood-based biomarkers associated with amyloid and tau pathology, neurodegeneration, and astrocyte activation show strong correlations with brain deposits of amyloid and tau in autopsy studies^4^, as well as with CSF and brain PET measures^5,6^. Emerging evidence indicates that these biomarkers have a fair-to-good performance in predicting cognitive decline, dementia, and their progression^7–14^. However, most studies have been carried out in clinical settings, focusing on select patients referred to memory clinics owing to cognitive complaints and/or deficits, offering limited evidence on the clinical validity of those biomarkers in the general population^15–17^. Population-based studies that include more diverse groups of individuals and have follow-up periods spanning many years are key for testing the clinical validity of these biomarkers in real-world contexts^18,19^.

In this community-based cohort study of more than 2,000 Swedish adults aged 60 and older included in the Swedish National study on Aging and Care in Kungsholmen (SNAC-K), who were followed for up to 16 years, we analyzed six AD blood biomarkers: the ratio of amyloid-β 42 to amyloid-β 40 (amyloid β42/40) and levels of p-tau 181, p-tau 217, total tau (t-tau), NfL, and GFAP. On the basis of a clinical assessment of dementia and AD, we aimed to: (1) estimate the hazard of all-cause and AD dementia in relation to baseline levels of AD biomarkers and (2) assess the biomarkers’ performance in the prediction of all-cause and AD dementia in a 10-year timeframe. This study aims to provide insights into the clinical validity of these biomarkers in the general population free from dementia at baseline.

## Results

### Baseline sample characteristics

During a 16-year follow-up period (median, 10.2 years; interquartile range, 5.11–11.92 years), 364 cases of dementia were identified (incidence rate (IR), 1.82; 95% confidence interval (CI), 1.64–2.01 per 100 person-years). Of them, 212 (58%) were cases of AD dementia (IR, 1.06; 95%, CI 0.92–1.21 per 100 person-years).

Out of the 2,290 participants without dementia at baseline and with available AD biomarkers, 142 (6%) withdrew from the study, resulting in a final sample of 2,148 participants with complete data. Those who withdrew were younger (mean age difference, –7.52 years; 95% CI, –9.27 to –5.76), more educated (47.2% had a university degree, compared with 35.4% of those retained), and were diagnosed with fewer chronic diseases (mean difference, –1.00 diseases; 95% CI, –1.39 to –0.60) than were those who remained in the study. The sex distribution was similar in both groups.

Baseline characteristics of the analytical sample (*n* = 2,148), categorized by whether dementia developed, are reported in Table 1. Participants who developed dementia were older, more likely to be female, less educated, diagnosed with more chronic diseases, and more likely to be apolipoprotein E allele ε4 (*APOE* *ε4*) carriers than those who did not develop dementia during the follow-up period.

**Table 1:** Baseline characteristics of study participants overall and by incident dementia

Characteristics	No dementia (*n* = 1784)	Incident dementia (*n* = 364)	Total (*n* = 2148)	*P*
Age (years, mean ± s.d.)	71.4 ± 10.4	79.8 ± 8.3	72.8 ± 10.6	<0.001
Female, *n* (%)	1,072 (60.0)	250 (68.7)	1,322 (61.6)	0.002
Education, *n* (%)				
Elementary	261 (14.6)	65 (17.9)	326 (15.2)	<0.001
High school	840 (47.1)	221 (60.7)	1,061 (49.4)	
University or above	682 (38.2)	78 (21.4)	760 (35.4)	
Chronic diseases, *n* (%)				
Number of chronic diseases	3.65 ± 2.4	4.78 ± 2.39	3.84 ± 2.39	<0.001
Hypertension	1,205 (67.5)	267 (73.4)	1,472 (68.5)	0.030
Ischemic heart diseases	222 (12.4)	77 (21.2)	299 (13.9)	<0.001
Heart failure	127 (7.1)	52 (14.3)	179 (8.3)	<0.001
Atrial fibrillation	135 (7.6)	49 (13.5)	184 (8.6)	<0.001
Cerebrovascular diseases	87 (4.9)	42 (11.5)	129 (6.0)	
Chronic kidney disease	532 (29.8)	186 (51.1)	718 (33.4)	<0.001
Anaemia	169 (9.5)	67 (18.4)	236 (11.0)	<0.001
Obesity	238 (13.3)	38 (10.4)	276 (12.9)	0.132
MMSE (mean ± s.d.)	28.8 ± 1.8	27.6 ± 2.4	28.6 ± 1.9	<0.001
Subjective memory complaints	1,181 (67.1)	286 (80.3)	1,467 (69.3)	<0.001
AD-type dementia, *n* (%)	N/A	212 (58.2)	212 (9.8)	
*APOE* *ε4* genotype, *n* (%)	474 (27.1)	140 (39.6)	614 (29.2)	<0.001

Missing: 1 in education; 4 in MMSE; 31 in subjective memory complaints; 46 in *APOE* genotype.

**Fig. 1:**
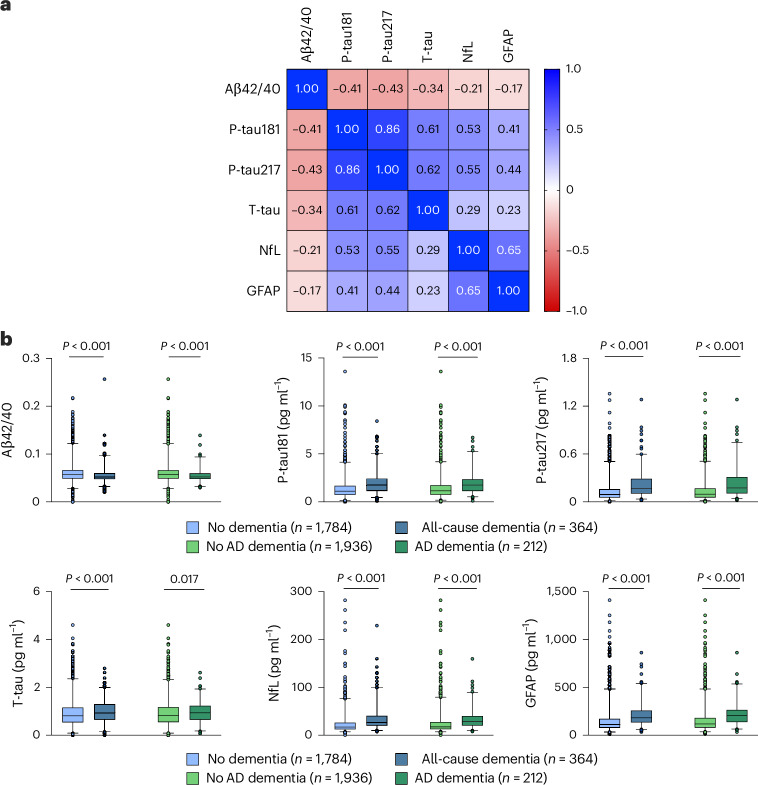
Blood biomarkers of Alzheimer's disease at baseline. **a**, Correlation matrix showing Spearman’s correlations between blood biomarkers of Alzheimer’s disease. **b**, Baseline biomarker levels by incident all-cause and AD dementia diagnosis. Box plots show the median (center line) and interquartile range (bounds of box) as well as the 2.5th and 97.5th percentiles (whiskers). *P* values were derived from a two-sided Mann–Whitney test.

Figure 1a shows the Spearman’s correlation matrix between the measured biomarkers. All biomarkers correlated with each other (*P* < 0.001). The strongest correlations were observed between p-tau217 and p-tau181 levels (Spearman’s rho = 0.86), NfL and GFAP levels (Spearman’s rho = 0.65), p-tau217 and t-tau levels (Spearman’s rho = 0.62), p-tau181 and t-tau levels (Spearman’s rho = 0.61), p-tau217 and NfL levels (Spearman’s rho = 0.55), and p-tau181 and NfL levels (Spearman’s rho = 0.53).

Figure 1b shows the baseline distribution of AD blood biomarkers by incident all-cause and AD dementia. Overall, individuals who developed dementia had lower baseline amyloid β42/40 and higher baseline levels of p-tau181, p-tau217, t-tau, NfL, and GFAP than those who did not develop all-cause and AD dementia.

### Biomarkers and incidence of all-cause and AD dementia

**Fig. 2:**
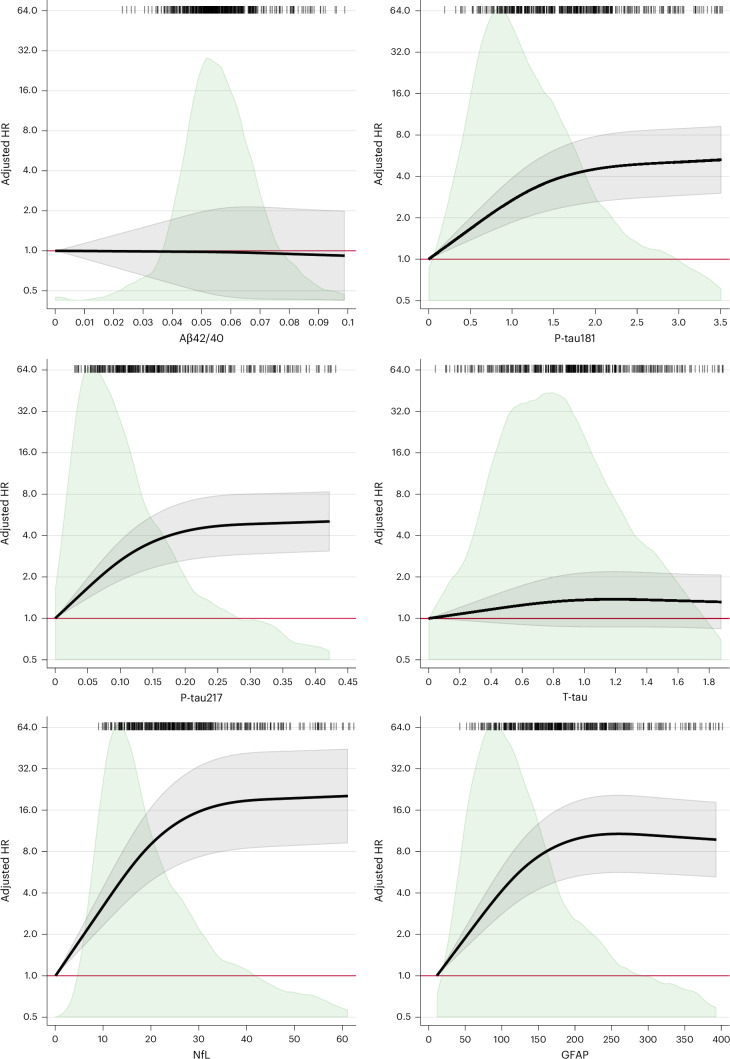
The association between baseline biomarker levels and hazard ratios for all-cause dementia with 95% confidence intervals, in the dementia-free analytical sample. Hazard ratios (HRs) are derived from Cox regression survival models. Models are adjusted for age; sex; education; disease status, including ischemic heart disease, atrial fibrillation, heart failure, cerebrovascular diseases, chronic kidney diseases, obesity, anaemia, and hypertension; and *APOE* genotype. Shaded green areas, the distribution of the biomarker levels in the entire population; spikes, incident dementia cases; black line, HR; gray shaded areas, confidence intervals; red line, the reference (HR = 1).

**Extended Data Fig. 1:**
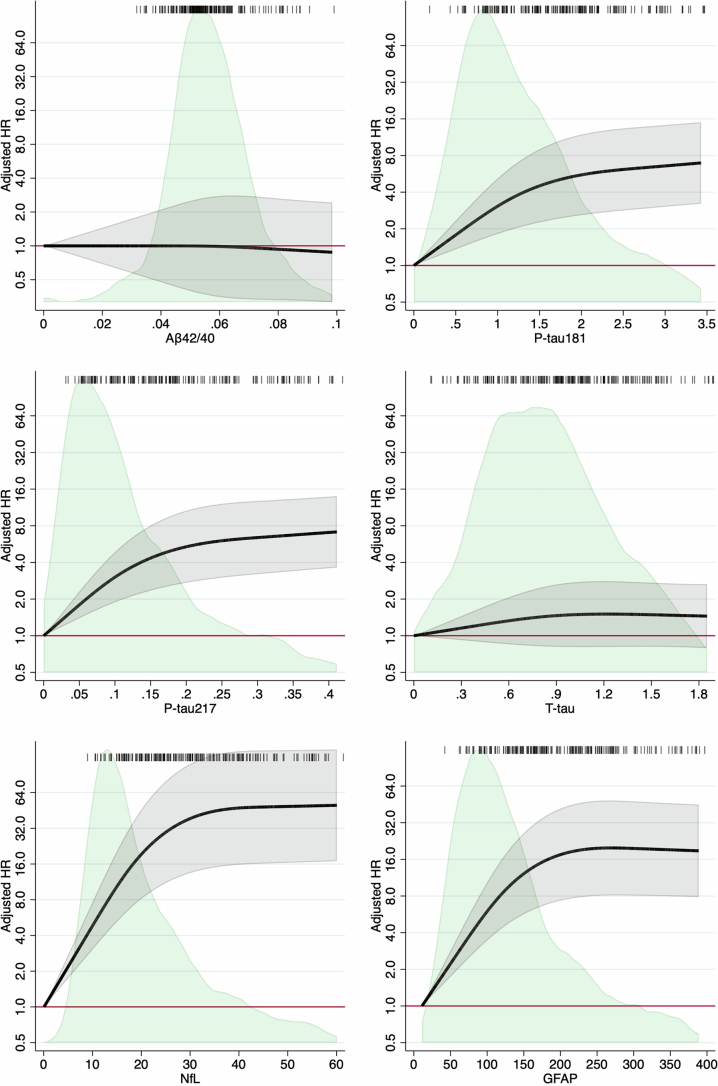
Association between baseline biomarker levels and hazard ratios (HR) for Alzheimer’s disease (AD) dementia with 95% confidence intervals, in the dementia-free analytical sample. HRs are derived from Cox regression survival models. Models are adjusted for age, sex, education, ischemic heart disease, atrial fibrillation, heart failure, cerebrovascular diseases, chronic kidney diseases, obesity, anaemia, hypertension, APOE genotype. Legend: Green shadow areas represent the distribution of the biomarker levels in the entire population, spikes the incident dementia cases, black line the HR, grey shadow areas confidence intervals, red line the reference (HR = 1).

We first investigated the associations between the raw concentration of individual biomarkers and all-cause and AD dementia, modeled through cubic splines to account for the non-linear association between biomarkers and dementia. As depicted in Figure 2, elevated baseline levels of p-tau181, p-tau217, NfL, and GFAP were associated with a higher hazard of developing all-cause dementia during the follow-up period, displaying a non-linear relationship. Similar results were observed for AD dementia (Extended Data Fig. 1).

Table 2 shows IRs and hazard ratios (HRs) for all-cause and AD dementia by quartiles of biomarkers levels, using the lower quartile as the reference. These analyses were performed using standardized biomarker concentrations to make direct comparisons of the magnitudes of biomarker–dementia associations. Fully adjusted models revealed a dose–response relationship (*P* < 0.001 for all) between baseline levels of p-tau181, p-tau217, NfL, and GFAP and higher hazards of all-cause and AD dementia. The third and fourth quartiles displayed a statistically significant association, particularly with AD dementia.

**Table 2:** Incidence rates and hazard ratios for all-cause and Alzheimer’s dementia with 95% confidence intervals by baseline biomarkers in the dementia-free analytical sample

	All-cause dementia		AD dementia	
Blood biomarkers	IR (95% CI) per 100 person-years	HR (95% CI)	IR (95% CI) per 100 person-years	HR (95% CI)
Aβ42 and Aβ40				
Q4 (ref.)^a^	1.04 (0.80–1.35)	1.00	0.56 (0.39–0.80)	1.00
Q3	1.39 (1.10–1.74)	0.96 (0.67–1.36)	0.76 (0.56–1.03)	0.96 (0.59–1.56)
Q2	2.64 (2.23–3.14)	1.41 (1.02–1.95)	1.68 (1.35–2.08)	1.76 (1.15–2.70)
Q1	2.34 (1.93–2.83)	1.12 (0.80–1.56)	1.32 (1.03–1.70)	1.21 (0.78–1.90)
*P* for trend		0.205		0.116
P-tau181				
Q1 (ref.)	0.60 (0.43–0.84)	1.00	0.24 (0.14–0.41)	1.00
Q2	1.21 (0.95–1.53)	1.38 (0.90–2.12)	0.74 (0.54–1.00)	2.00 (1.11–3.96)
Q3	1.89 (1.54–2.29)	1.50 (1.01–2.26)	1.17 (0.91–1.50)	2.25 (1.22–4.17)
Q4	4.40 (3.78–5.12)	2.51 (1.68–3.74)	2.57 (2.11–3.13)	3.69 (2.01–6.78)
*P* for trend		<0.001		<0.001
P-tau217				
Q1 (ref.)	0.5 (0.35–0.73)	1.00	0.26 (0.16–0.43)	1.00
Q2	1.13 (0.89–1.47)	1.81 (1.15–2.84)	0.66 (0.48–0.93)	1.81 (0.98–3.33)
Q3	2.08 (1.73–2.51)	2.29 (1.49–3.51)	1.15 (0.89–1.47)	1.97 (1.10; 3.53)
Q4	4.41 (3.78–5.14)	3.31 (2.15–5.09)	2.71 (2.23–3.30)	3.27 (1.83–5.86)
*P* for trend		<0.001		<0.001
T-tau				
Q1 (ref.)	1.37 (1.09–1.73)	1.00	0.79 (0.58–1.07)	1.00
Q2	1.27 (1.00–1.60)	0.88 (0.56–1.09)	0.72 (0.53–0.99)	0.80 (0.52–1.24)
Q3	2.27 (1.89–2.74)	1.14 (0.84–1.54)	1.43 (1.13–1.81)	1.21 (0.81–1.79)
Q4	2.49 (2.08–3.00)	1.13 (0.83–1.53)	1.36 (1.06–1.75)	1.27 (0.85–1.89)
*P* for trend		0.124		0.076
NfL				
Q1 (ref.)	0.42 (0.28–0.63)	1.00	0.22 (0.13–0.39)	1.00
Q2	0.84 (0.63–1.11)	1.10 (0.67–1.82)	0.46 (0.32–0.67)	1.13 (0.58–2.22)
Q3	2.38 (1.99–2.85)	2.13 (1.33–3.45)	1.26 (0.98–1.61)	2.28 (1.19–4.34)
Q4	4.87 (4.19–5.65)	3.29 (1.97–5.48)	3.08 (2.56–3.72)	5.01 (2.55–9.88)
*P* for trend		<0.001		<0.001
GFAP				
Q1 (ref.)	0.44 (0.29–0.65)	1.00	0.17 (0.09–0.33)	1.00
Q2	0.98 (0.75–1.27)	1.55 (0.94–2.56)	0.51 (0.35–0.73)	1.87 (0.89–3.89)
Q3	2.27 (1.89–2.73)	2.49 (1.56–3.98)	1.27 (0.99–1.63)	3.25 (1.63–6.47)
Q4	4.42 (3.81–5.13)	3.52 (2.18–5.68)	2.84 (2.35–3.42)	5.65 (2.82–11.31)
*P* for trend		<0.001		<0.001

HRs are derived from Cox regression survival models. Cox regression models are adjusted for age; sex; education; disease diagnoses, including ischemic heart disease, atrial fibrillation, heart failure, cerebrovascular disease, chronic kidney disease, obesity, anemia, and hypertension; and *APOE* genotype. AD, Alzheimer's disease dementia; IR, incidence rates; CI, confidence interval; HR, hazard ratio; ref., reference group; Q, quartile. ^a^For the Aβ42 to Aβ40 ratio, the highest quartile (Q4) was chosen as the reference.

#### Subgroup analyses

Supplementary Tables 1 and 2 show HRs for all-cause and AD dementia by biomarkers levels in groups stratified by age, sex, and *APOE* genotype. Concerning age, the associations between all-cause and AD dementia and levels of p-tau181, p-tau217, t-tau, NfL, and GFAP were stronger in participants younger than 78 than in those aged 78 and older. In sex-stratified analyses, the associations between the dementia outcomes and p-tau181, p-tau217, and NfL levels were stronger in women, but the association between dementia outcomes and GFAP levels was stronger in men. Finally, the hazard for all-cause and AD dementia was higher in *APOE ε4* carriers in the highest quartile of p-tau217 and GFAP levels and in non-*APOE-ε4* carriers in the highest quartile of NfL levels. In the subgroup of those with memory complaints, for p-tau217, NfL and GFAP we obtained consistent and overall stronger findings (Supplementary Table 3).

#### Sensitivity analyses

When we repeated the analyses after excluding those with a baseline Mini-Mental State Examination (MMSE) score below 27 (*n* = 228), we obtained attenuated but consistent association estimates (Supplementary Table 4). Supplementary Table 5 shows the HRs for all-cause and AD dementia stratified by biomarker levels, after restricting the follow-up time to the first six years of observation. The survival analyses indicate that HRs are overall stronger in this period than in the entire follow-up period; however, the CIs for AD dementia are wide owing to the limited number of cases and their uneven distribution across different groups (AD dementia cases by NfL quartiles: Q1, 1; Q2, 5; Q3, 24; Q4, 68). The results were consistent even after incorporating inverse probability weighting for dropout status in the models (Supplementary Table 6).

### Biomarkers and prediction of all-cause and AD dementia

**Fig. 3:**
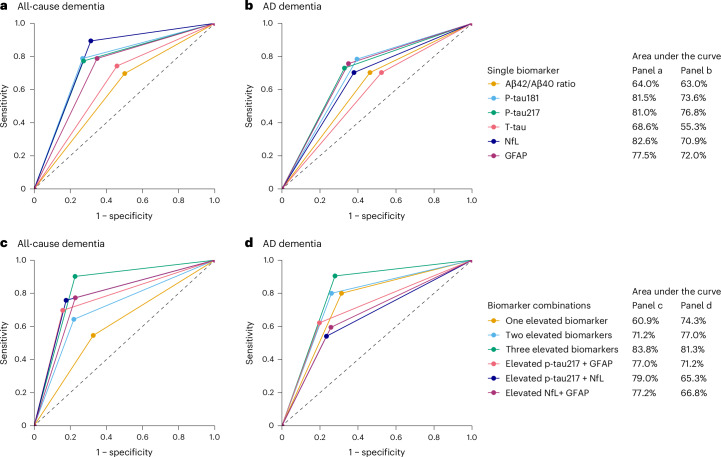
Blood biomarkers of Alzheimer's disease and prediction of all-cause and Alzheimer's disease dementia. **a**–**d**, Areas under the curves for all-cause (**a**,**c**) and Alzheimer’s disease (**b**,**d**) dementia, by baseline AD blood biomarker level for single biomarkers and biomarker combinations.

We randomly divided the sample into a training set (80% of the study population) and a testing set (20%), and used a bootstrapping approach on the training set to determine the optimal raw biomarker cut-off that maximized Youden’s index for predicting all-cause and AD dementia over a 10-year period (Supplementary Table 7). The identified cut-offs were tested in the unseen portion of the dataset. Figure 3 and Table 3 show the areas under the curves (AUCs), along with their 95% CIs, and the diagnostic performance measures for cumulative incidence of all-cause and AD dementia over a 10-year period. The best AUCs for all-cause and AD dementia were achieved by levels of the two p-tau isoforms, NfL, and GFAP. AUCs ranged from 77.5% for GFAP to 82.6% for NfL for all-cause dementia and from 70.9% for NfL to 76.8% for p-tau217 for AD dementia. The best performance measure obtained was the negative predictive values (NPVs), which were always greater than 90% for all biomarkers.

**Table 3:** Predictive performance measures of blood biomarkers of Alzheimer’s disease for detecting 10-year all-cause and AD dementia

		AUC (95% CI)	Correctly classified participants (accuracy) (95% CI)	Sensitivity (95% CI)	Specificity (95% CI)	PPV (95% CI)	NPV (95% CI)
		**All-cause dementia**					
**Biomarker**	**Cut-off**						
Aβ42/40	0.057	64.0 (56.6–71.2)	52.9 (48.1– 57.6)	69.7 (58.3–80.7)	49.9 (44.7–55.0)	19.7 (14.9–25.1)	90.3 (86.1–94.2)
P-tau181	1.512 pg ml^–1^	81.5 (76.1–86.4)	74.0 (69.9–78.4)	78.8 (68.3–88.1)	73.2 (68.8–77.6)	34.2 (26.5–41.7)	95.1 (92.4–97.5)
P-tau217	0.134 pg ml^–1^	81.0 (75.5–86.0)	73.4 (69.3–77.5)	77.3 (66.7–87.2)	72.6 (68.1–77.2)	33.3 (26.1–41.0)	94.8 (92.1–97.2)
T-tau	0.832 pg ml^–1^	68.6 (61.4–75.8)	57.2 (52.9–61.9)	74.2 (63.6–84.8)	54.2 (49.3–59.1)	22.3 (16.8–27.9)	92.2 (88.6–95.7)
NfL	20.171 pg ml^−1^	82.6 (77.6–87.1)	71.8 (67.7–75.9)	89.4 (81.3–96.3)	68.6 (63.9–73.4)	33.5 (26.6–40.8)	97.3 (95.2–99.2)
GFAP	142.515 pg ml^–1^	77.5 (71.4–83.1)	67.2 (62.9–71.5)	78.8 (68.8–88.2)	65.2 (60.3–69.9)	28.6 (22.2–35.4)	94.6 (91.6–97.2)
		**AD dementia**					
**Biomarker**	**Cut-off**						
Aβ42/40	0.056	63.0 (54.3–71.5)	55.2 (50.4–60.0)	70.3 (54.3–84.6)	53.7 (48.7–58.8)	12.9 (8.5–17.6)	94.9 (91.7–97.7)
P-tau181	1.410 pg ml^–1^	73.6 (66.5–80.5)	62.1 (57.6–66.7)	78.4 (64.4–91.1)	60.5 (55.7–65.4)	16.2 (11.1–21.8)	96.6 (94.1–98.7)
P-tau217	0.143 pg ml^–1^	76.8 (69.4–83.8)	67.6 (63.3–71.9)	73.0 (58.1–86.7)	67.1 (62.6–71.7)	17.8 (12.0–24.0)	96.2 (93.8–98.4)
T-tau	0.846 pg ml^–1^	55.3 (46.7–63.7)	49.6 (44.8–54.7)	70.3 (55.0–84.4)	47.6 (42.7–52.7)	11.6 (7.7–16.0)	94.3 (90.7–97.3)
NfL	21.399 pg ml^–1^	70.9 (62.6–78.6)	62.8 (58.3–67.4)	70.3 (55.3–84.6)	62.1 (57.4–67.1)	15.3 (10.3–21.1)	95.6 (92.8–98.0)
GFAP, pg ml^–1^	148.737 pg ml^–1^	72.0 (63.9–79.8)	66.0 (61.4–70.5)	75.7 (61.3–89.3)	65.0 (60.3–69.7)	17.4 (11.8–23.4)	96.5 (94.1–98.5)

#### Combination of biomarkers

Figure 3 shows the predictive performance for detecting future all-cause and AD dementia over 10 years, on the basis of elevated levels of several biomarkers, namely p-tau217, NfL, and GFAP. The presence of elevated values of one, two, or three elevated biomarkers yielded AUC values of 60.9%, 71.1%, and 83.8% for all-cause dementia, respectively, and 74.3%, 77.0%, and 81.3% for AD dementia, respectively. When we investigated specific pairs of biomarkers, elevated levels of both p-tau217 and NfL demonstrated the best performance in predicting all-cause dementia (AUC, 79.0%) with a PPV of 43.1% and NPV of 95.1%. Elevated levels of both p-tau217 and GFAP showed the highest performance in predicting AD dementia (AUC, 71.2%) with a PPV of 23.5% and NPV of 95.6%. Predictive performance measures for the combinations of different biomarkers are reported in Supplementary Table 8. When repeating the AUCs (and 95% CI) and the diagnostic performance measures for all-cause and AD dementia in the subgroup reporting memory complaints, we obtained similar findings (Supplementary Table 9).

## Discussion

In this community-based study, we found that cognitively intact older adults with higher levels of p-tau181, p-tau217, NfL, and GFAP face a higher risk of developing both all-cause and Alzheimer's dementia than do those with lower biomarker levels, showing a non-linear dose–response relationship. Elevated concentrations of p-tau181, p-tau217, NfL, and GFAP demonstrated an overall good predictive performance for the occurrence of all-cause and AD dementia over the next 10 years. These biomarkers displayed high sensitivity, high NPVs, and low PPVs. We also observed improved prediction for all-cause and AD dementia by combining p-tau217 with either NfL or GFAP, respectively, which led to improved positive predictive values. Although this result suggests that AD blood biomarkers are not yet suitable screening tools for cognitively unimpaired older adults living in community settings, it does not diminish their potential for ruling out impending dementia.

Clinical studies have repeatedly shown a robust correlation between AD blood biomarkers and brain deposits of amyloid and tau^20–22^, as well as good accuracy in predicting cognitive decline and dementia onset^7,11,23^. However, there is limited evidence regarding their performance in community settings^24,25^. Our findings are in line with previous evidence from the few population-based longitudinal studies examining the association between dementia risk and the levels of p-tau181, NfL, and GFAP^14,16,17^. We further enhance this evidence by incorporating p-tau217 and providing estimates for both associations and predictive performance.

The most-studied biomarkers are those specifically associated with AD pathology^20,25,26^. However, our study revealed weak associations between blood amyloid β42/40 and incident dementia. Although this finding might seem counterintuitive, several considerations need to be made. First, brain-derived molecules tend to exist in lower concentrations in the blood, particularly for amyloid. Studies have consistently demonstrated that Aβ concentrations can be up to tenfold lower in blood than in CSF^2^. Second, substantial production of amyloid molecules, particularly Aβ42, occurs in the periphery, for example by the liver, skeletal muscle system, and arteries (particularly in presence of atherosclerosis), meaning that only a small fraction reflects brain pathology^27^. Consequently, the relationship between Aβ in the blood and neuropathological changes is not that specific. Finally, mass spectrometry, as opposed to immunoassay technology, could offer greater accuracy in quantifying blood levels of amyloid^28^, but it is difficult to scale and unaffordable in community settings. These observations partly apply also to blood t-tau^29^, for which our study revealed no statistically significant associations with dementia onset.

On the contrary, in our study, we observed strong associations between baseline blood levels of p-tau181 and p-tau217 and future all-cause and AD dementia. This aligns with findings from previous studies conducted in clinical settings^5,7,11,22^ and two population-based studies^14,17^. The blood p-tau level reflects not only tau pathology, but also—and more strongly—brain deposits of amyloid^30,31^. Of note, p-tau217 has been recognized as a standalone biomarker for AD diagnosis in the latest criteria from the Alzheimer’s Association Workgroup, published in July 2024 (ref. ^32^); this is in line with our findings, which revealed a stronger association when we restricted our analysis to only AD dementia. Even after excluding individuals with a MMSE score below 27, the results, although somewhat attenuated, remained largely consistent. These findings, coupled with the performance results described below, reinforce the notion that altered p-tau levels are a powerful biomarker for dementia and AD pathology, appearing early in the disease process and preceding the onset of noticeable cognitive symptoms.

We also found that high levels of NfL and GFAP in circulation are associated with an increased risk of all-cause and AD dementia. NfL is a non-specific marker of loss of axonal integrity^23,33,34^, and we found that altered levels of NfL appeared many years before disease detection, suggesting it has a role even in early disease stages. In line with our findings, Lu et al., in a recent study based on the Atherosclerosis Risk in Communities cohort in the United States, found that elevated NfL levels showed the strongest association with the risk of all-cause dementia^17^. Few studies have explored the levels of blood GFAP in AD so far^35–37^. Elevated GFAP levels are indicative of abnormal activation of the astrocytes that surround amyloid plaques and are frequently altered in AD dementia^32^. Our findings, in line with others^38^, confirm the crucial role of inflammation and astrocytes activation in dementia development.

In this study, we also examined the predictive performance of each biomarker and found that p-tau181, p-tau217, NfL, and GFAP demonstrated good performance in detecting both all-cause and AD dementia, with AUCs ranging between 71% and 83%. However, comparing our findings directly with existing literature is challenging owing to the scarcity of population-based studies that report both positive and negative predictive measures. A study involving 1,525 individuals living in community settings who had a mean age of 58 years, part of the ARIC cohort, found that high levels of p-tau181, NfL, and GFAP, measured using Simoa technology, predicted future all-cause dementia, with AUCs ranging from 62% to 69% (for NfL)^17^. Additionally, two studies that enrolled primary-care patients have been conducted. Stocker et al., in a sample of 768 individuals aged 50–75 who were recruited by general practitioners, reported AUCs ranging from 61% to 73% for the predicting future dementia using elevated p-tau181, GFAP, and NfL levels over a mean follow-up period of 10 years^14^. A previous study conducted in 1,213 primary-care patients, including 44% with mild cognitive impairment and 33% with overt dementia, tested the ability of AD biomarkers measured by mass spectrometry to predict AD pathology and clinical AD^13^. When clinical AD (corroborated by CSF and PET tests) was used as primary outcome, the diagnostic accuracy of p-tau217 was as high as 91%, with a PPV of 86% and a NPV of 98%. However, a substantial lack of knowledge remains regarding the PPVs and NPVs of AD blood biomarkers in the prediction of clinical (AD) dementia incidence in primary care.

We enhanced the current evidence on biomarker accuracy in the community by including p-tau217 in our analysis. This biomarker demonstrated strong performance in predicting future all-cause (AUC, 81%) and AD (AUC, 77%) dementia. The good predictive performance detected in our study was driven by the high negative prediction (more than 90% for all six biomarkers). The PPVs for single-biomarker analyses were low, not exceeding 30%, which is possibly attributable to the small proportion of individuals in our study setting who developed dementia, as the study population primarily consisted mainly of cognitively intact individuals with at most minimal memory complaints. However, improved prediction of dementia was obtained by combining different biomarkers, with the PPVs reaching 43% in the case of all-cause dementia, while maintaining high NPVs. This enhancement was particularly evident when p-tau217 was combined with NfL for all-cause dementia prediction, or with GFAP for AD dementia prediction.

These findings prompt several clinical considerations regarding the potential utility of AD blood biomarkers outside specialized settings. First, the high NPVs obtained from single-biomarker analyses suggest that, in community settings, AD blood biomarkers have the potential to rule out impending dementia over a 10-year period, assuming other risk factors remain stable. This finding could help counsel individuals concerned about their cognitive health, providing reassurance about low dementia risk, enhancing psychosocial well-being, and reducing unnecessary healthcare interventions. However, the high NPVs were coupled with low PPVs. These results, if confirmed in future studies, suggest that these biomarkers used alone and in individuals without any clinical symptoms are not yet suitable screening tools. This is in line with the latest diagnostic criteria for AD^32^, which caution against testing asymptomatic individuals for AD biomarkers. Our results reinforce these recommendations.

Key strengths of the current study include the large population-based sample and the follow-up period of up to 16 years, which included extensive in-person evaluations for both diagnosing dementia and assessing comorbidities. These features enabled us to adjust our analyses for multiple covariates and demonstrate the associations between blood biomarkers with dementia, independent of potential confounders. Dementia diagnoses followed a standardized three-step process involving two specially trained physicians, overseen by a senior neurologist external to the data-collection team who is an expert in cognitive assessment and dementia diagnosis. This rigorous diagnostic approach is a distinctive feature of our population study. Of note, we also identified cases of dementia among people who died during the follow-up period and did not have a prior diagnosis of dementia by reviewing clinical charts and death registers, thus reducing the risk of death obscuring the presence of dementia. Yet, some milder cases might not have been captured, leading to a possible underestimation of the association. Finally, we had the opportunity to analyse an extensive panel of AD biomarkers, including p-tau217, in a population-based setting in relation to incident dementia.

Some limitations need to be acknowledged. First, in the SNAC-K, all-cause and AD dementia diagnoses are purely clinical and do not involve the use of neuroimaging or CSF biomarkers. Although we are confident in our (all-cause) dementia diagnoses derived from the described procedure, relying exclusively on clinical data for identifying dementia subtypes poses a risk of misclassification. However, most dementia cases detected in community settings occur in people older than 75. As such, dementia cases are rarely pure AD cases; a mixed pathology is the most common neuropathological feature^39,40^. Second, in our study, AD biomarkers were measured in serum, which can affect their bioavailability compared with plasma. For instance, amyloid β levels, like other protein biomarkers, can be more stable in plasma owing to the anticoagulants used during collection^41^, which prevents, among other things, clotting and degradation that could interfere with measurement accuracy^42,43^. Third, previous studies^30,44–46^ conducted in clinical settings and based on plasma samples have demonstrated the superiority of certain assays, different from those used in our study, in predicting AD pathology. Fourth, to investigate biomarkers’ predictive performance, we derived specific cut-offs from a training sample and tested them in an unseen segment of the dataset. Future studies are needed to validate our findings in separate cohorts, because we did not conduct an external validation of these cut-offs. Despite finding good to excellent predictive performance for dementia prediction using these cut-offs, establishing definitive thresholds is outside the scope of this study. The investigation of biomarker cut-offs remains ongoing, and it is premature to propose cut-offs for clinical use on the basis of our findings. Fifth, we faced a considerable amount of baseline missing data for biomarker analyses. Individuals with missing data were generally older, less educated, and had a higher burden of comorbidities than those who provided blood samples. As a result, we expect that these individuals were at a higher risk of dementia, which could result in our findings being an underestimation.

In conclusion, in this study based on data from more than 2,000 cognitively intact community-dwelling older adults, we found that high levels of p-tau181, p-tau217, NfL, and GFAP were associated with an increased risk of developing all-cause or AD dementia over a mean follow-up of 10 years, independent of a wide range of potential confounders. These biomarkers also showed good predictive performance, particularly in ruling out impending dementia, owing to their high NPVs. At the same time, the low PPVs achieved underscore that a single biomarker is insufficient for screening purposes, particularly among cognitively unimpaired community-dwelling older adults. Future research integrating multiple biomarkers and focusing on at-risk subgroups might help enhance early detection of dementia. Such future efforts should also account for diversity across different socioeconomic backgrounds and ethnic groups. Tailoring biomarker-based strategies to individual clinical profiles will be crucial in optimizing counselling, care, and intervention in community settings.

## Methods

### Study population

This study is based on data from the SNAC-K, an ongoing longitudinal population-based study including individuals aged 60 and older residing in the Kungsholmen district of central Stockholm^47^. In the initial assessment phase (2001–2004), a total of 3,363 participants were evaluated, yielding a response rate of 73.3%. Subsequently, participants have undergone regular follow-ups every 6 years for the younger age cohorts (60–78 years) and every 3 years for the older age cohorts (≥78 years).

**Extended Data Fig. 2:**
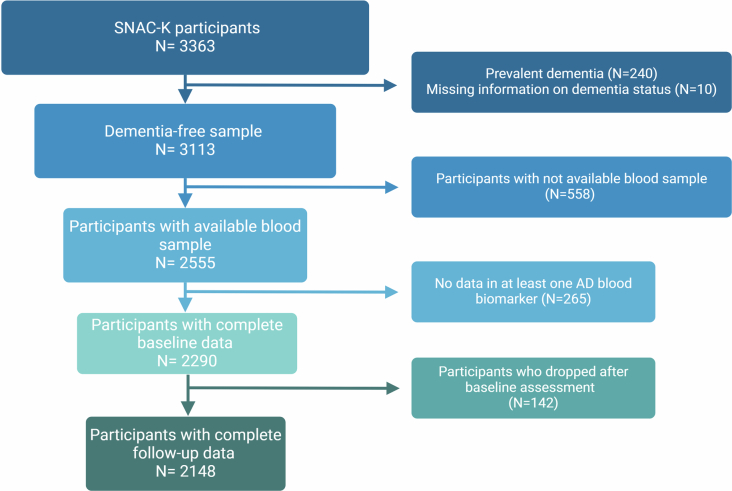
Flow chart of the study participation. Created in BioRender. https://BioRender.com/l73u834.

The study participation flowchart is presented in Extended Data Figure 2. From the initial cohort of SNAC-K participants at baseline (*n* = 3,363), we excluded individuals diagnosed with dementia (*n* = 240) and those with missing information on dementia status (*n* = 10). Of these, 2,555 participants consented to blood sampling, which included a comprehensive health assessment (for example, hemoglobin, C-reactive protein, and cholesterol levels) and collected additional samples. Among the collected blood samples, we excluded those from participants with missing data for at least one AD blood biomarker (*n* = 265). Participants with missing biomarker data (*n* = 823) were older, were more likely to be female, were less educated, and had a higher burden of chronic diseases than those with complete data (*P* < 0.001 for all comparisons). Out of the 2,290 dementia-free individuals with available biomarker data, 142 dropped out after baseline assessment, leaving a final analytical sample of 2,148 individuals.

All participants provided written informed consent for their involvement in the study, with proxy consent obtained for those who were cognitively impaired. The research protocol for each phase of the SNAC-K study received approval from the Regional Ethical Review Board in Stockholm (Dnrs: KI 01-114, 04-929/3, Ö26-2007, 2009/595-32, 2010/447-31/2, 2013/828-31/3, and 2016/730-31/1), and ethical standards of the Declaration of Helsinki were followed throughout the investigation.

The results of this study are reported in keeping with the Strengthening the Reporting of Observational Studies in Epidemiology recommendations^48^.

### Data collection

At each study visit, data were collected through standardized procedures, including face-to-face interviews and clinical and laboratory examinations conducted by trained physicians, nurses, and psychologists. Participants were evaluated either at the Stockholm Gerontology Research Center or, for those unable to reach it, at home or at the institution.

During the interview with a nurse, demographic information such as age, sex, and education level was obtained. Educational attainment was categorized into elementary school, high school, and college or university or above. Venous blood samples were obtained for DNA extraction, and genotyping was performed to identify *APOE* alleles. Participants were subsequently categorized as either *ε4* carriers or *ε4* non-carriers. Memory complaints were assessed through a question during the physician’s interview: “Do you think your memory has worsened?” Participants who replied “yes, somewhat” and “yes, a lot” were classified as having memory complaints.

To ensure that we obtained a comprehensive understanding of participants’ health status, a thorough clinical procedure was implemented, which has been described elsewhere^49^. This involved medical history collection during physician interviews, clinical (both general and neurological) examinations, diagnostic tests (both instrumental and blood tests), and data from inpatient and outpatient records, medical journals, and the Swedish National Patient Register. Diagnoses were coded according to the International Classification of Diseases, 10th revision, following a clinical review conducted by trained physicians.

#### Alzheimer’s disease blood biomarkers

**Extended Data Fig. 3:**
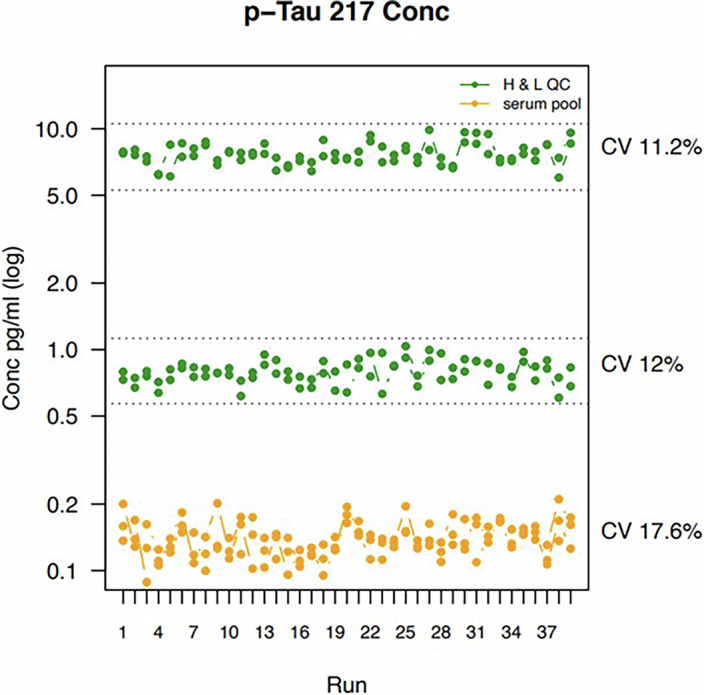
P-tau217 assay precision. P-Tau 217 levels measured in the commercial control 1, control 2 and pool of sera obtained in the analysis of the SNAC-K cohort (2366 samples divided in 39 runs). Between run CV is indicated for serum pool, control 1 (low) and control 2 (high).

Peripheral venous blood samples were collected at baseline. Fasting was not compulsory, and 95% (*n* = 2,041) of the participants did not fast. Blood samples were centrifuged, and serum aliquots were stored at the Bio Bank of Karolinska Institutet at −80 °C in Cryovial PK100 tubes until analysis. The analyses were conducted at the Affinity Proteomics Stockholm Unit (SciLifeLab), using the Quanterix SR-X (software version 1.2.0) Biomarker Detection System. Serum concentrations of NfL and GFAP were measured using the Simoa Neuro 2-plex B Kit (Quanterix, cat. no. 103520). The Simoa Neuro 3-plex A Kit (Quanterix, cat. no. 101995) was used to measure serum Aβ40, Aβ42, and t-tau levels, and the Simoa p-tau181 Advantage V2 Kit (Quanterix, cat. no. 103714)) was used to measure serum p-tau181 levels. The commercial assay Simoa ALZpath p-tau217 Advantage PLUS (Quanterix, cat. no. 104570), developed for the Quanterix HD-X system, was used to quantify p-tau217. Before analyzing the samples, we assessed the compatibility of the kits with our SR-X system and performed serum validation, as described more in detail in the following section and in Supplementary Table 10 and Extended Data Figure 3. For each kit, 25 μl of sample was diluted 1:4, and assays were performed according to the manufacturer’s instructions or internal validation protocols. The Quanterix instrument calculates average enzyme per bead values for calibrators, controls, and samples across all proteins. The Quanterix SR-X software automatically performs curve-fitting and concentration extrapolation, generating graphical representations using the calibrators—known concentration series of an analyte—and a four-parameter logistic curve fit. Precision was estimated for each assay. The within-run coefficient of variation (CV) was calculated using a triplicate serum pool (from SNAC-K samples), a control 1 and a control 2 from the Quanterix kits included in each run. The average CV for all runs is reported in Supplementary Table 11. The between-run CV was determined on the basis of concentration values for the serum pool, control 1, and control 2 across all runs. An overview of the precision assessment is available in Supplementary Table 11 and Extended Data Figure 3. Data below limit of detection were replaced, using a not missing at random strategy and single-value imputation, with a value of 0 assigned (number of imputed measurements: 6 for Aβ42, 15 for p-tau181, 5 for p-tau217, and 15 for t-tau).

### Simoa ALZpath p-Tau 217 validation in serum and compatibility with SR-X

The Simoa ALZpath p-Tau 217 Advantage PLUS was initially developed for the Quanterix HD-X platform. Recently, assay compatibility on the Quanterix SR-X platform has been demonstrated by the company. However, in both platforms (HD-X and SR-X), analytical validation was performed only in EDTA plasma. In this study, we assessed assay compatibility and performed a validation in serum. Assay validation and compatibility were performed using the kit lot no. 504180, and samples were run using the kit lot no. 504307. A summary of tests performed, including values for each analytical parameter tested, is reported in Supplementary Table 10.

In brief, lyophilized calibrator and controls 1 (low) and 2 (high), included in the kit, were reconstituted according to the lot-specific certificate of analysis using, respectively, Calibrator Diluent or Control Diluent. The limit of detection was calculated as 2.5 s.d. above the background (mean of calibrator blanks). Analytical LLOQ was the lowest calibrator with a CV < 20% and mean recovery of 80–120% over all runs. The lower limit of quantification (LLOQ) was calculated as: analytical LLOQ × required dilution. For the spike and recovery tests, calibrator diluent, commercial human normal serum (BioIVT, cat. no. HUMANSRMPNN; lot or sample nos. HMN696994–HMN696996), and a pooled sample from the SNAC-K cohort were spiked with one of two concentrations of p-tau217 (1 pg ml^–1^ and 5 pg ml^–1^) and with 5% commercial human normal CSF (Lee Biosolutions, cat. no. 991-19-S, lot no. T5647).

For the dilution linearity, normal serum and EDTA plasma were diluted twice serially, and the average percentage recovery is reported for each sample. We opted for a 1:4 dilution for serum, because it yielded higher recovery than did the suggested 1:3 dilution for plasma.

All tests were conducted in triplicate on the same plate and day over a span of 3 days. Assay precision was evaluated both within and between runs. The average CV within and between runs was determined for the SNAC-K serum pool, control 1, and control 2 during the validation experiments, as well as across the 39 runs conducted for the full cohort (Supplementary Tables 10 and 11 and Extended Data Fig. 3).

During assay validation, control 1 and control 2 always fell within the range specified in the lot-specific certificate of analysis. Subsequently, while analyzing the 39 plates, we defined our range on the basis of observed trends: control 1 ranged from 0.570 to 1.124 pg ml^–1^ and control 2 ranges from 5.272 to 10.528 pg ml^–1^ (Extended Data Fig. 3).

#### All-cause and Alzheimer’s dementia diagnosis

The clinical diagnosis of dementia was performed in keeping with the *Diagnostic and Statistical Manual of Mental Disorders, Fourth Edition* criteria^50^, and the diagnosis of AD dementia followed the NINCDS-ADRDA criteria^51^. Dementia diagnosis was based on a comprehensive assessment with medical history—including comorbidities—and drug history, as well as a general and neurological examination, including an ad hoc examination for Parkinson’s disease and parkinsonisms. Physicians evaluated cognitive functioning through questions covering problem-solving, abstract thinking, self- and time-space orientation, and general knowledge, as well as the MMSE, a clock drawing test, digit span forward and backward, and a short story assessment of frontal-lobe function. Additionally, the independence of daily living, both basic and instrumental, was assessed. Final diagnoses were made through a three-step process. Initially, the examining physician who directly interacted with the participant provided a preliminary diagnosis. Subsequently, a second preliminary diagnosis was made by a reviewing physician, blind to the first diagnosis, on the data-collection team. In instances of discordance between the initial and secondary diagnoses, a final diagnosis was determined by senior neurologists, who are experts in cognitive assessment and dementia and not directly involved in the data-collection process. For individuals who died between follow-up examinations, clinical charts along with their death certificates were collected and reviewed by the same physicians involved in the initial diagnostic process^52^.

### Statistical analyses

In a first stage, to assess departure from a simple linear trend, we modelled the continuous raw biomarkers using restricted cubic splines with three knots at fixed percentiles (25th, 50th, and 75th percentiles) of their distribution. Next, blood biomarkers were standardized into *z*-scores on the basis of the sample’s mean and s.d. to facilitate comparison between coefficients. Standardization was based on the whole sample with available blood-biomarkers at baseline. In the survival analyses, each biomarker was analyzed as a categorical variable on the basis of quartiles. Cox models were applied to estimate the associations of each biomarker with all-cause and AD dementia. The timescale was defined as the follow-up duration since baseline (2001–2004), and follow-up times were defined for each individual as the time of all-cause or AD dementia diagnosis, withdrawal from the study, or death, whichever came first. The proportional hazard assumption was assessed by regressing the scaled Schoenfeld’s residuals against survival time. No deviation from the proportional hazard assumption was detected. In the analyses focused on AD, individuals diagnosed with other dementias or who died without dementia were censored.

Potential confounding factors were identified a priori on the basis of a literature review and available data from the study population. Data included age; sex; educational level; disease status, including ischemic heart disease, atrial fibrillation, heart failure, cerebrovascular disease, chronic kidney disease, obesity, anaemia, and hypertension; and *APOE* *ε4* status (non-carriers or carriers).

#### Subgroup analysis

For the incidence of all-cause and AD dementia, stratified analyses by age groups (<78 years and ≥78 years), sex, and *APOE* *ε4* allele status were conducted. Additionally, we repeated the analyses only in those who reported memory complaints.

#### Sensitivity analyses

The following sensitivity analyses were conducted: (1) we repeated the analyses by excluding those with a MMSE score below 27 at baseline (*n* = 228); (2) we repeated the survival analyses after restricting the follow-up period to the first 6 years; and (3) to assess whether loss to follow-up (*n* = 142) might affect the results, we used a logistic regression to estimate dropout probability based on age, sex, education, number of chronic diseases, and biomarker levels at baseline, and an inverse probability weight (IPW) was generated. We then repeated the main analyses by adding the IPWs in the Cox proportional hazards model.

A non-parametric bootstrapping approach was used to determine the optimal cut-off for each biomarker^53^. This method evaluated all cut points and selected the cut point that maximized Youden’s index within each bootstrap sample. The mean cutpoint across the 5,000 bootstrap samples was chosen as the optimal cut-off. We first calculated optimal cut-offs for 10-year all-cause and AD dementia. For internal validation of the classification performance for the biomarkers, the original dataset was randomly split into a training data set and a testing data set, with a split ratio of 80:20. Performance methods were reported for both training and testing datasets.

We further tested the performance of the following combinations of biomarkers with elevated levels defined using the cut-offs: (1) one elevated biomarker, (2) two elevated biomarkers, (3) three elevated biomarkers, (4) elevated p-tau217 and NfL, (5) elevated p-tau217 and GFAP, and (6) elevated NfL and GFAP.

Additionally, we evaluated the performance of the biomarkers in individuals who reported memory complaints.

A two-tailed *P* < 0.05 was considered statistically significant in all analyses. The statistical analyses were performed using Stata version 17 (StataCorp) and R version 4.3.3 (The R Foundation for Statistical Computing); GraphPad Prism 9 was used to generate graphical representations.

### Reporting summary

Further information on research design is available in the Nature Portfolio Reporting Summary linked to this article.

## Online content

Any methods, additional references, Nature Portfolio reporting summaries, source data, extended data, supplementary information, acknowledgements, peer review information; details of author contributions and competing interests; and statements of data and code availability are available at 10.1038/s41591-025-03605-x.

## Supplementary information

Supplementary Information Supplementary Tables 1–11.

Reporting Summary

## Data Availability

SNAC-K data are sensitive data; thus, they cannot be shared publicly, but raw and analyzed de-identified data can be requested by qualified researchers at https://www.snac-k.se/. The request will be reviewed to ensure confidentiality and intellectual-property obligations. A data-sharing agreement must be signed prior to data release.

## Code availability

No custom code or mathematical algorithm was employed in the study. Analysis scripts for this study are available at https://github.com/axinaxia/blood-based-biomarker-study.git.
