# Supplementary material for: Blood-based biomarkers of Alzheimer’s disease and incident dementia in the community
Source: Nat Med. 2025 Mar 26;31(6):2027–35. doi: 10.1038/s41591-025-03605-x (PMC12176656; doi:10.1038/s41591-025-03605-x)
Supplement: Supplementary file 1 — Supplementary Tables 1–11. [file 41591_2025_3605_MOESM1_ESM.pdf]

nature medicine

Supplementary information

https://doi.org/10.1038/s41591-025-03605-x

Blood-based biomarkers of Alzheimer's disease and incident dementia in the community

In the format provided by the authors and unedited

Supplementary files.

Supplementary Table 1. Hazard ratios (HR) for **all-cause dementia** with 95% confidence intervals (CIs) by baseline biomarkers in the dementia-free analytical sample, by age, sex and APOEe4

	**Age**		**Sex**		**APOE4 genotype****	
	**Age <78 years (N=1412)**	**Age 78 + years (N=878)**	**Male (N=880)**	**Female (N=1410)**	**No ε4 alleles (N=1568)**	**At least one ε4 allele (N=653)**
**Biomarkers**						
**Aβ42/Aβ40**						
Q4 (Ref*)	1.00	1.00	1.00	1.00	1.00	1.00
Q3	0.97 (0.52-1.84)	0.99 (0.64-1.52)	0.88 (0.48-1.62)	0.98 (0.63-1.51)	0.91 (0.59-1.40)	1.24 (0.67-2.31)
Q2	**2.00 (1.13-3.53)**	1.15 (0.78-1.71)	1.26 (0.73-2.19)	1.32 (0.88-1.99)	1.12 (0.74-1.69)	**1.76 (1.02-3.01)**
Q1	0.78 (0.40-1.55)	1.12 (0.76-1.66)	0.80 (0.45-1.42)	1.22 (0.80-1.84)	0.98 (0.64-1.51)	1.29 (0.74-2.26)
**P-tau181**						
Q1 (Ref.)	1.00	1.00	1.00	1.00	1.00	1.00
Q2	1.04 (0.56-1.96)	1.73 (0.95-3.15)	1.24 (0.59-2.63)	1.48 (0.88-2.52)	1.58 (0.89-2.80)	1.21 (0.62-2.37)
Q3	1.36 (0.73-2.53)	1.47 (0.83-2.59)	1.51 (0.76-3.02)	1.47 (0.88-2.45)	**1.87 (1.08-3.25)**	1.19 (0.64-2.23)
Q4	**3.13 (1.62-6.05)**	**2.44 (1.41-4.21)**	1.71 (0.85-3.45) [#]	**3.05 (1.85-5.01)** [#]	**2.69 (1.56-4.64)**	**2.68 (1.45-4.98)**
**P-tau217**						
Q1 (Ref.)	1.00	1.00	1.00	1.00	1.00	1.00
Q2	1.79 (0.90-3.54)	1.66 (0.89-3.10)	0.77 (0.34-1.70) [#]	**2.32 (1.32-4.07)** [#]	**2.25 (1.26-4.02)**	1.11 (0.51-2.40)
Q3	**2.17 (1.10-4.28)**	1.60 (0.90-2.85)	1.09 (0.53-2.23) [#]	**2.53 (1.47-4.36)** [#]	**2.30 (1.31-4.04)**	1.50 (0.75-3.00)
Q4	**5.48 (2.72-11.02)** [#]	**1.96 (1.11-3.46)** [#]	2.01 (0.98-4.14)	**3.29 (1.90-5.71)**	**2.58 (1.45-4.60)**	**3.41 (1.72-6.76)**
**T-tau**						
Q1 (Ref)	1.00	1.00	1.00	1.00	1.00	1.00
Q2	1.03 (0.55-1.92)	0.74 (0.49-1.11)	0.99 (0.56-1.74)	0.71 (0.46-1.09)	0.74 (0.47-1.17)	0.90 (0.53-1.53)
Q3	1.73 (0.95-3.15)	1.09 (0.76-1.56)	1.03 (0.60-1.78)	1.22 (0.84-1.78)	1.30 (0.88-1.93)	1.07 (0.65-1.75)

Q4	**2.08 (1.13-3.83)**	0.92 (0.64-1.31)	0.95 (0.51-1.78)	1.18 (0.82-1.70)	1.02 (0.68-1.52)	1.46 (0.88-2.41)
**NfL**						
Q1 (Ref.)	1.00	1.00	1.00	1.00	1.00	1.00
Q2	1.13 (0.61-2.10)	0.83 (0.33-2.11)	1.20 (0.55-2.61)	1.17 (0.60-2.27)	1.17 (0.51-2.69)	1.13 (0.60-2.13)
Q3	**2.53 (1.32-4.84)**	1.32 (0.57-3.08)	2.08 (0.98-4.41)	**2.50 (1.33-4.70)**	**2.68 (1.23-5.83)**	**1.97 (1.04-3.76)**
Q4	**3.53 (1.59-7.86)**	2.00 (0.86-4.68)	**3.20 (1.39-7.41)**	**3.75 (1.92-7.32)**	**4.73 (2.10-10.63)** #	**2.25 (1.10-4.58)** #
**GFAP**						
Q1 (Ref.)	1.00	1.00	1.00	1.00	1.00	1.00
Q2	**2.09 (1.02-4.28)**	1.03 (0.50-2.14)	**2.40 (1.14-5.06)**	1.09 (0.55-2.14)	1.17 (0.61-2.25)	**2.20 (1.01-4.82)**
Q3	**4.00 (2.02-7.94)**	1.27 (0.64-2.51)	**4.14 (2.03-8.42)**	1.83 (0.97-3.47)	**1.93 (1.05-3.56)**	**3.19 (1.52-6.70)**
Q4	**7.36 (3.49-15.48)** #	1.80 (0.92-3.51) #	**5.44 (2.62-11.32)**	**2.50 (1.31-4.78)**	**2.60 (1.38-4.91)**	**4.85 (2.28-10.29)**

HRs are derived from Cox regression survival models.

Models are adjusted for age, sex, education, ischemic heart disease, atrial fibrillation, heart failure, cerebrovascular diseases, chronic kidney diseases, obesity, anaemia, hypertension, APOE genotype.

*For the Aβ42/Aβ40 ratio the highest quartile (Q4) was chosen as reference
**69 missing values in APOE genotype
p for interaction <0.05

Supplementary Table 2. Hazard ratios (HR) for **AD dementia** with 95% confidence intervals (CIs) by baseline biomarkers in the dementia-free analytical sample, by age, sex and APOEe4

	**Age**		**Sex**		**APOE4 genotype****	
	**Age <78 years (N=1412)**	**Age 78+ years (N=878)**	**Male (N=880)**	**Female (N=1410)**	**No ε4 alleles (N=1568)**	**At least one ε4 allele (N=653)**
**Biomarkers**						
**Aβ42/Aβ40**						
Q4 (Ref*)	1.00	1.00	1.00	1.00	1.00	1.00
Q3	1.08 (0.46-2.53)	0.95 (0.53-1.72)	0.97 (0.38-2.46)	0.93 (0.53-1.63)	0.88 (0.47-1.67)	1.34 (0.63-2.85)
Q2	**2.35 (1.09-5.05)**	1.43 (0.85-2.40)	2.12 (0.96-4.65)	1.45 (0.86-2.43)	1.43 (0.80-2.57)	**1.96 (1.02-3.77)**
Q1	0.76 (0.30-1.95)	1.22 (0.72-2.07)	1.19 (0.52-2.74)	1.12 (0.65-1.92)	1.22 (0.66-2.24)	1.15 (0.57-2.32)
**P-tau181**						
Q1 (Ref.)	1.00	1.00	1.00	1.00	1.00	1.00
Q2	1.49 (0.62-3.58)	**3.05 (1.16-8.03)**	2.67 (0.71-10.02)	1.95 (0.94-4.04)	1.15 (0.57-2.32)	1.58 (0.63-4.00)
Q3	1.90 (0.79-4.57)	**2.70 (1.06-6.86)**	**4.02 (1.16-13.90)**	1.71 (0.83-3.53)	**3.22 (1.24-8.36)**	2.02 (0.85-4.77)
Q4	**5.00 (2.00-12.54)**	**4.08 (1.63-10.21)**	**3.78 (1.07-13.31)**	**3.78 (1.87-7.62)**	**4.45 (1.72-11.48)**	**4.55 (1.93-10.74)**
**P-tau217**						
Q1 (Ref.)	1.00	1.00	1.00	1.00	1.00	1.00
Q2	1.89 (0.73-4.87)	1.57 (0.69-3.58)	0.77 (0.23-2.51)	**2.32 (1.12-4.82)**	**3.52 (1.44-8.59)** #	0.65 (0.24-1.77) #
Q3	**2.65 (1.05-6.67)**	1.38 (0.64-2.98)	1.33 (0.47-3.75)	**2.25 (1.10-4.62)**	**2.77 (1.14-6.75)** #	1.32 (0.58-3.04) #
Q4	**7.57 (2.93-19.56)** #	2.05 (0.97-4.32) #	2.61 (0.92-7.36)	**3.57 (1.75-7.30)**	**3.45 (1.41-8.47)**	**3.63 (1.62-8.16)**
**T-tau**						
Q1 (Ref)	1.00	1.00	1.00	1.00	1.00	1.00
Q2	0.99 (0.43-2.30)	0.73 (0.43-1.24)	1.00 (0.46-2.16)	0.71 (0.41-1.23)	1.03 (0.55-1.92)	0.66 (0.34-1.28)
Q3	2.04 (0.91-4.53)	1.08 (0.68-1.71)	1.09 (0.52-2.25)	1.23 (0.76-1.99)	1.73 (0.99-3.02) #	0.86 (0.47-1.56) #

Q4	**2.45 (1.10-5.45)**	0.89 (0.56-1.42)	1.13 (0.49-2.62)	1.17 (0.73-1.89)	1.18 (0.65-2.11)	1.37 (0.77-2.45)
**NfL**						
Q1 (Ref.)	1.00	1.00	1.00	1.00	1.00	1.00
Q2	1.00 (0.43-2.32)	1.15 (0.32-4.15)	0.51 (0.17-1.52) #	2.27 (0.84-6.13) #	2.10 (0.45-9.69)	0.97 (0.43-2.16)
Q3	**2.91 (1.24-6.80)**	1.40 (0.42-4.61)	1.13 (0.43-2.95) #	**4.45 (1.68-11.77)** #	3.82 (0.86-16.84)	**2.48 (1.13-5.41)**
Q4	**7.13 (2.72-18.67)**	2.97 (0.90-9.86)	**3.39 (1.23-9.30)** #	**8.67 (3.17-23.71)** #	**11.83 (2.66-52.73)**#	**3.30 (1.39-7.79)** #
**GFAP**						
Q1 (Ref.)	1.00	1.00	1.00	1.00	1.00	1.00
Q2	2.78 (0.94-8.20)	1.13 (0.41-3.13)	**4.09 (1.10-15.25)**	1.07 (0.44-2.60)	1.31 (0.46-3.73)	2.47 (0.87-6.99)
Q3	**6.34 (2.31-17.36)**	1.34 (0.51-3.49)	**9.33 (2.68-32.54)**	1.75 (0.76-4.03)	2.41 (0.90-6.47)	**4.01 (1.50-10.69)**
Q4	**13.24 (4.50-38.93)**	2.45 (0.97-6.22)	**12.17 (3.44-43.15)**	**3.31 (1.44-7.63)**	**3.91 (1.43-10.68)**	**7.22 (2.69-19.38)**

HRs are derived from Cox regression survival models.

Models are adjusted for age, sex, education, ischemic heart disease, atrial fibrillation, heart failure, cerebrovascular diseases, chronic kidney diseases, obesity, anaemia, hypertension, APOE genotype.

*For the Aβ42/Aβ40 ratio the highest quartile (Q4) was chosen as reference
**69 missing values in APOE genotype
p for interaction <0.05

Supplementary Table 3. Incidence rates (IR) per 100 person/year and hazard ratios (HR) for **all-cause (n = 286) and Alzheimer´s dementia (AD) (n = 169)** with 95% confidence intervals (CIs) by baseline biomarkers in the dementia-free analytical sample considering only participants **with memory complaints at baseline.**

	**All-cause dementia**		**AD dementia**	
	**IR (95%CI)**	**HR (95%CI)**	**IR (95%CI)**	**HR (95%CI)**
**Biomarkers**				
**Aβ42/Aβ40**				
Q4 (Ref*)	1.14 (0.84-1.56)	1.00	0.63 (0.41-0.95)	1.00
Q3	1.54 (1.18-2.00)	0.93 (0.61-1.41)	0.88 (0.62-1.24)	0.99 (0.57-1.72)
Q2	3.16 (2.61-3.83)	1.38 (0.94-2.03)	2.04 (1.60-2.59)	**1.74 (1.06-2.88)**
Q1	2.79 (2.26-3.45)	1.04 (0.70-1.55)	1.56 (1.17-2.07)	1.11 (0.65-1.89)
P for trend		0.495		0.387
**P-tau181**				
Q1 (Ref.)	0.64 (0.43-0.97)	1.00	0.25 (0.13-0.48)	1.00
Q2	1.45 (1.11-1.90)	1.56 (0.94-2.58)	0.91 (0.64-1.27)	2.53 **(1.19-5.35)**
Q3	2.30 (1.86-2.86)	**2.08 (1.29-3.33)**	1.50 (1.15-1.96)	**3.42 (1.67-7.00)**
Q4	4.71 (3.96-5.61)	**2.86 (1.78-4.60)**	2.71 (2.15-3.41)	**4.25 (2.07-8.74)**
P for trend		**<0.001**		**<0.001**
**P-tau217**				
Q1 (Ref.)	0.58 (0.38-0.88)	1.00	0.29 (0.16-0.52)	1.00
Q2	1.27 (0.95-1.71)	**2.04 (1.19-3.47)**	0.81 (0.56-1.17)	2.31 (1.14-4.68)
Q3	2.59 (2.11-3.18)	**2.86 (1.74-4.70)**	1.46 (1.12-1.92)	**2.99 (1.54-5.83)**
Q4	4.74 (3.99-5.64)	**3.53 (2.13-5.86)**	2.89 (2.31-3.61)	**4.06 (2.07-7.94)**
P for trend		**<0.001**		**<0.001**
**T-tau**				
Q1 (Ref)	1.48 (1.13-1.93)	1.00	0.89 (0.63-1.26)	1.00
Q2	1.64 (1.26-2.12)	0.91 (0.62-1.34)	0.89 (0.63-1.27)	0.81 (0.49-1.34)
Q3	2.57 (2.08-3.18)	1.24 (0.87-1.77)	1.63 (1.25-2.13)	1.24 (0.79-1.95)
Q4	2.90 (2.36-3.57)	1.36 (0.95-1.95)	1.66 (1.26-2.18)	1.39 (0.88-2.20)
P for trend		0.045		0.069
**NfL**				
Q1 (Ref.)	0.45 (0.28-0.73)	1.00	0.25 (0.13-0.49)	1.00
Q2	1.10 (0.81-1.48)	1.58 (0.88-2.85)	0.63 (0.42-0.93)	1.66 (0.76-3.63)
Q3	2.68 (2.19-3.27)	**3.05 (1.73-5.36)**	1.49 (1.14-1.95)	**3.27 (1.55-6.91)**
Q4	5.26 (4.43-6.25)	**4.63 (2.52-8.48)**	3.29 (2.65-4.09)	**6.57 (2.98-14.50)**
P for trend		**<0.001**		**<0.001**
**GFAP**				

Q1 (Ref.)	0.58 (0.38-0.88)	1.00	0.18 (0.09-0.39)	1.00
Q2	1.16 (0.86-1.57)	**1.73 (1.00-3.01)**	0.61 (0.40-0.93)	**2.65 (1.12-6.25)**
Q3	2.61 (2.12-3.23)	**2.64 (1.58-4.43)**	1.59 (1.22-2.08)	**4.60 (2.05-10.33)**
Q4	4.86 (4.10-5.75)	**3.94 (2.32-6.69)**	3.13 (2.54-3.86)	**8.00 (3.54-18.09)**
P for trend		**<0.001**		**<0.001**

HRs are derived from Cox regression survival models, adjusted for age, sex, education, ischemic heart disease, atrial fibrillation, heart failure, cerebrovascular diseases, chronic kidney diseases, obesity, anaemia, hypertension, APOE genotype.

*For the Aβ42/Aβ40 ratio the highest quartile (Q4) was chosen as reference

Supplementary Table 4. Hazard ratios (HR) for **all-cause and Alzheimer´s dementia** with 95% confidence intervals (CIs) by baseline biomarkers in the dementia-free analytical sample **excluding those with a MMSE<27 (n=228)**

	**All-cause dementia**		**AD dementia**	
	**IR (95%CI)**	**HR (95%CI)**	**IR (95%CI)**	**HR (95%CI)**
<u>**Biomarkers**</u>				
**Aβ42/Aβ40**				
Q4 (Ref*)	0.90 (0.68-1.20)	1.00	0.50 (0.34-0.73)	1.00
Q3	1.14 (0.88-1.47)	0.90 (0.60-1.33)	0.60 (0.42-0.86)	0.85 (0.50-1.46)
Q2	2.00 (1.63-2.46)	1.27 (0.88-1.83)	1.30 (1.01-1.68)	1.59 (0.99-2.55)
Q1	1.73 (1.38-2.19)	1.03 (0.70-1.50)	1.04 (0.77-1.40)	1.09 (0.66-1.81)
P for trend		0.475		0.261
**P-tau181**				
Q1 (Ref.)	0.52 (0.36-0.75)	1.00	0.22 (0.13-0.39)	1.00
Q2	1.10 (0.85-1.42)	1.56 (0.98-2.48)	0.68 (0.49-0.94)	**2.19 (1.13-4.27)**
Q3	1.46 (1.16-1.84)	**1.58 (1.01-2.47)**	0.83 (0.61-1.13)	**2.01 (1.04-3.87)**
Q4	3.27 (2.71-3.95)	**2.78 (1.79-4.32)**	2.07 (1.64-2.62)	**3.90 (2.05-7.41)**
P for trend		**<0.001**		**<0.001**
**P-tau217**				
Q1 (Ref.)	0.48 (0.33-0.70)	1.00	0.25 (0.15-0.42)	1.00
Q2	1.00 (0.76-1.32)	**1.72 (1.06-2.77)**	0.60 (0.42-0.86)	1.83 (0.96-3.49)
Q3	1.68 (1.36-2.08)	**2.05 (1.30-3.23)**	0.89 (0.67-1.19)	**1.98 (1.07-3.66)**
Q4	3.26 (2.69-3.95)	**2.87 (1.81-4.56)**	2.14 (1.69-2.71)	**3.42 (1.85-6.31)**
P for trend		**<0.001**		**0.001**
**T-tau**				
Q1 (Ref)	1.10 (0.85-1.43)	1.00	0.61 (0.43-0.87)	1.00
Q2	0.97 (0.73-1.28)	0.80 (0.54-1.18)	0.61 (0.43-0.87)	0.91 (0.55-1.50)
Q3	1.70 (1.36-2.13)	1.12 (0.78-1.59)	1.11 (0.85-1.46)	1.19 (0.75-1.89)
Q4	2.01 (1.62-2.48)	1.38 (0.97-1.96)	1.07 (0.80-1.44)	1.40 (0.88-2.23)
P for trend		0.055		0.164
**NfL**				
Q1 (Ref.)	0.38 (0.24-0.58)	1.00	0.20 (0.11-0.36)	1.00
Q2	0.77 (0.57-1.04)	1.24 (0.73-2.11)	0.40 (0.27-0.61)	1.20 (0.58-2.52)
Q3	2.09 (1.71-2.55)	**2.70 (1.62-4.50)**	1.12 (0.85-1.47)	**2.97 (1.48-5.97)**
Q4	3.53 (2.91-4.27)	**3.88 (2.23-6.74)**	2.42 (1.92-3.05)	**6.56 (3.16-13.62)**
P for trend		**<0.001**		**<0.001**
**GFAP**				
Q1 (Ref.)	0.45 (0.30-0.66)	1.00	0.18 (0.10-0.33)	1.00
Q2	0.84 (0.63-1.12)	1.48 (0.89-2.48)	0.43 (0.28-0.64)	1.76 (0.83-3.74)
Q3	1.77 (1.43-2.20)	**2.41 (1.49-3.90)**	1.01 (0.75-1.34)	**3.00 (1.48-6.07)**

Q4	3.41 (2.85-4.10)	**3.60 (2.19-5.93)**	2.33 (1.87-2.90)	**6.04 (2.97-12.29)**
P for trend		**<0.001**		**<0.001**

HRs are derived from Cox regression survival models, adjusted for age, sex, education, ischemic heart disease, atrial fibrillation, heart failure, cerebrovascular diseases, chronic kidney diseases, obesity, anaemia, hypertension, APOE genotype.

*For the Aβ42/Aβ40 ratio the highest quartile (Q4) was chosen as reference

Supplementary Table 5. Incidence rates (IR) per 100 person/year and hazard ratios (HR) for all-cause (n = 161) and Alzheimer´s dementia (AD) (n = 89) with 95% confidence intervals (CIs) by baseline biomarkers in the dementia-free analytical sample **considering only the first six years of follow-up**.

	**All-cause dementia**		**AD dementia**	
	**IR (95%CI)**	**HR (95%CI)**	**IR (95%CI)**	**HR (95%CI)**
**Biomarkers**				
**Aβ42/Aβ40**				
Q4 (Ref*)	0.51 (0.31-	1.00	0.20 (0.09-	1.00
Q3	0.93 (0.64-	1.38 (0.73-2.62)	0.51 (0.31-	2.03 (0.78-5.29)
Q2	2.09 (1.63-	**1.88 (1.05-3.39)**	1.24 (0.89-	**3.20 (1.32-7.75)**
Q1	2.16 (1.67-	**1.79 (1.00-3.21)**	1.19 (0.84-	**2.77 (1.14-6.75)**
P for trend		**0.044**		0.029
**P-tau 181**				
Q1 (Ref.)	0.24 (0.11-	1.00	0.07 (0.02-	1.00
Q2	0.69 (0.45-	2.12 (0.89-5.05)	0.46 (0.27-	**5.14 (1.15-22.99)**
Q3	1.30 (0.94-	**2.41 (1.05-5.49)**	0.78 (0.52-	**5.59 (1.30-24.12)**
Q4	3.75 (3.07-	**4.50 (2.02-9.99)**	1.98 (1.50-	**9.81 (2.32-41.42)**
P for trend		**<0.001**		**0.001**
**P-tau 217**				
Q1 (Ref.)	0.16 (0.07-	1.00	0.10 (0.03-	1.00
Q2	0.62 (0.39-	**3.13 (1.15-8.48)**	0.45 (0.26-	**3.93 (1.11-13.92)**
Q3	1.38 (1.01-	**3.76 (1.46-9.66)**	0.71 (0.46-	3.40 (0.99-11.63)
Q4	3.84 (3.14-	**5.79 (2.28-**	2.06 (1.57-	**5.94 (1.78-19.75)**
P for trend		**<0.001**		**0.008**
**T-tau**				
Q1 (Ref)	0.67 (0.43-	1.00	0.40 (0.23-	1.00
Q2	0.88 (0.60-	1.15 (0.63-2.10)	0.44 (0.26-	1.03 (0.47-2.29)
Q3	1.94 (1.49-	**1.98 (1.17-3.36)**	1.20 (0.86-	**2.03 (1.03-4.03)**
Q4	2.24 (1.74-	**1.71 (1.01-2.87)**	1.12 (0.78-	1.60 (0.81-3.18)
P for trend		**0.029**		0.111
**NfL**				
Q1 (Ref.)	0.10 (0.03-	1.00	0.03 (0.01-	1.00
Q2	0.23 (0.11-	1.59 (0.41-6.18)	0.13 (0.05-	2.99 (0.33-26.91)
Q3	1.55 (1.15-	**5.70 (1.71-**	0.76 (0.50-	**10.55 (1.38-80.83)**
Q4	4.22 (3.49-	**8.09 (2.36-**	2.50 (1.90-	**21.31 (2.74-**
P for trend		**<0.001**		**<0.001**
**GFAP**				
Q1 (Ref.)	0.20 (0.09-	1.00	0.07 (0.02-	1.00
Q2	0.44 (0.25-	1.61 (0.57-4.56)	0.20 (0.09-	2.05 (0.41-10.23)
Q3	1.60 (1.20-	**3.13 (1.21-8.13)**	0.84 (0.56-	**4.74 (1.08-20.72)**
Q4	3.67 (3.01-	**4.13 (1.58-**	2.18 (1.68-	**8.07 (1.85-35.19)**
P for trend		**<0.001**		**<0.001**

HRs are derived from Cox regression survival models, adjusted for age, sex, education, ischemic heart disease, atrial fibrillation, heart failure, cerebrovascular diseases, chronic kidney diseases, obesity, anaemia, hypertension, APOE genotype.

*For the Aβ42/Aβ40 ratio the highest quartile (Q4) was chosen as reference

Supplementary Table 6. Hazard ratios (HR) for all-cause and Alzheimer´s dementia (AD) with 95% confidence intervals (CIs) by baseline biomarkers in the dementia-free analytical sample with **inverse probability weighting**

	**All-cause**	**AD dementia**
	**HR (95%CI)**	**HR (95%CI)**
**Biomarkers**		
**Aβ42/Aβ40**		
Q4 (Ref*)	1.00	1.00
Q3	0.98 (0.69-1.39)	0.99 (0.62-1.59)
Q2	1.35 (0.98-1.85)	**1.66 (1.11-2.47)**
Q1	1.10 (0.79-1.53)	1.16 (0.75-1.78)
P for trend	0.319	0.212
**P-tau 181**		
Q1 (Ref.)	1.00	1.00
Q2	1.37 (0.90-2.09)	**2.06 (1.12-3.81)**
Q3	**1.50 (1.00-2.25)**	**2.23 (1.24-4.01)**
Q4	**2.51 (1.67-3.77)**	**3.65 (2.03-6.56)**
P for trend	**<0.001**	**<0.001**
**P-tau217**		
Q1 (Ref.)	1.00	1.00
Q2	**1.73 (1.11-2.70)**	1.80 (0.98-3.32)
Q3	**1.98 (1.30-3.02)**	**1.97 (1.11-3.51)**
Q4	**2.85 (1.84-4.42)**	**3.29 (1.83-5.94)**
P for trend	**<0.001**	**<0.001**
**T-tau**		
Q1 (Ref)	1.00	1.00
Q2	0.78 (0.56-1.10)	0.78 (0.50-1.20)
Q3	1.14 (0.83-1.56)	1.17 (0.78-1.76)
Q4	1.13 (0.83-1.54)	1.17 (0.78-1.75)
P for trend	0.128	0.163
**NfL**		
Q1 (Ref.)	1.00	1.00
Q2	1.12 (0.68-1.85)	1.14 (0.59-2.23)
Q3	**2.19 (1.35-3.55)**	**2.32 (1.21-4.46)**
Q4	**3.36 (2.01-5.61)**	**5.10 (2.62-9.93)**
P for trend	**<0.001**	**<0.001**
**GFAP**		
Q1 (Ref.)	1.00	1.00
Q2	1.55 (0.93-2.57)	1.86 (0.87-3.95)

Q3	**2.51 (1.55-4.05)**	**3.26 (1.59-6.68)**
Q4	**3.55 (2.16-5.85)**	**5.68 (2.75-11.73)**
P for trend	**<0.001**	**<0.001**

HRs are derived from Cox regression survival models adjusted for age, sex, education, ischemic heart disease, atrial fibrillation, heart failure, cerebrovascular diseases, chronic kidney disease, obesity, anaemia, hypertension, APOE genotype.

*For the Aβ42/Aβ40 ratio the highest quartile (Q4) was chosen as reference.

Supplementary Table 7. Predictive performance measures of blood biomarkers of AD for detecting 10-year all-cause and AD dementia in the **training set in the SNAC-K cohort**

		**Training set**					
		**All-cause dementia**					
		**AUC (95%CI)**	**Correctly classified participants (accuracy) (95%CI)**	**Sensitivity (95%CI)**	**Specificity (95%CI)**	**PPV (95%CI)**	**NPV (95%CI)**
**Biomarker**	**Cutoff**						
Aβ42/40	0.057	58.1 (54.2;62.0)	53.3 (51.1;55.6)	68.9 (62.5;75.3)	51.3 (48.8;53.7)	15.6 (13.2; 17.9)	92.7 (90.8; 94.4)
P-tau 181, pg/mL	1.512	69.3 (65.6;73.0)	67.3 (65.0;69.4)	62.6 (55.9;69.1)	67.9 (65.6;70.1)	20.3 (17.1; 23.4)	93.3 (91.7; 94.7)
P-tau 217, pg/mL	0.134	72.6 (69.0;76.1)	67.9 (65.8-70.1)	69.5 (63.0;75.8)	67.7 (65.5;70.0)	21.9 (18.8;25.2)	94.4 (93.0;95.8)
T-tau, pg/mL	0.832	54.4 (50.4;58.4)	52.1 (49.8;54.3)	62.1 (55.4;68.9)	50.7 (48.3;53.1)	14.1 (11.9;16.6)	91.1 (89.1;93.0)
NfL, pg/mL	20.171	75.7 (72.7;78.8)	64.1 (61.9;66.3)	78.8 (72.9;84.4)	62.2 (59.8;64.6)	21.4 (18.5;24.4)	95.7 (94.5;96.9)
GFAP, pg/mL	142.515	74.1 (71.0;77.3)	66.9 (64.8;69.1)	73.4 (67.2;79.4)	66.1 (63.7;68.4)	22.1 (19.0;25.2)	95.0 (93.7;96.3)
		**AD dementia**					
Aβ42/40	0.056	58.9 (53.9;63.6)	52.3 (50.0;54.7)	69.6 (60.9;78.0)	51.1 (48.6;53.5)	9.3 (7.4;11.2)	95.9 (94.5;97.2)
P-tau 181, pg/mL	1.410	71.5 (66.9;76.0)	63.4 (61.2;65.8)	66.1 (57.4;74.5)	63.2 (60.9;65.6)	11.5 (9.1;13.9)	96.3 (95.1;97.4)
P-tau 217, pg/mL	0.143	74.7 (70.3;79.0)	68.9 (66.8;71.2)	73.2 (65.1;81.2)	68.6 (66.4;71.0)	14.4 (11.6;17.3)	97.3 (96.3;98.2)

T-tau, pg/mL	0.846	57.9 (52.6;63.1)	53.2 (50.8;55.7)	67.0 (57.9;75.2)	52.3 (49.8;54.8)	9.2 (7.3;11.1)	95.7 (94.2;97.0)
NfL, pg/mL	21.399	78.0 (74.5;81.3)	64.2 (61.9;66.5)	85.7 (79.0;91.8)	62.6 (60.2;65.1)	14.2 (11.6;16.8)	98.38 (97.6;99.1)
GFAP, pg/mL	148.737	77.0 (72.9;80.7)	66.1 (63.8;68.4)	75.9 (67.7;83.7)	65.4 (63.0;67.7)	13.6 (11.0;16.3)	97.4 (96.4;98.3)

Supplementary Table 8. Predictive performance measures of different combinations of blood biomarkers of AD for detecting 10-year all-cause and AD dementia

		AUC (95%CI)	Correctly classified participants (accuracy) (95%CI)	Sensitivity (95%CI)	Specificity (95%CI)	PPV (95%CI)	NPV (95%CI)
		**All-cause dementia**					
**Number of altered biomarkers**	1 biomarker	60.9 (45.8-76.4)	66.8 (61.1-72.1)	54.6 (25.0-85.7)	67.3 (61.5-72.9)	6.4 (2.1-11.6)	97.3 (94.7-99.5)
	2 biomarkers	71.2 (57.3-83.9)	77.2 (71.5-82.1)	64.3 (37.5-88.9)	78.0 (72.5-83.0)	15.0 (6.5-24.5)	97.3 (94.7-99.5)
	3 biomarkers	83.78 (78.5-88.4)	79.7 (74.7-84.2)	90.2 (81.3-97.9)	77.4 (71.9-82.7)	46.5 (36.4-56.4)	97.3 (94.7-99.5)
**Combinations of biomarkers**	**P-tau 217 + NfL**	79.0 (73.4-84.3)	81.3 (77.7-84.9)	75.8 (65.0-85.9)	82.3 (78.5-86.2)	43.1 (34.3-52.4)	95.1 (92.6-97.3)
	**P-tau 217+ GFAP**	76.9 (71.3-82.8)	82.0 (78.6-85.7)	69.7 (58.9-80.7)	84.2 (80.5-87.8)	43.8 (34.7-53.7)	94.0 (91.3-96.5)
	**GFAP+ NfL**	77.2 (71.7-82.5)	77.2 (73.5-81.1)	77.3 (67.1-87.1)	77.2 (72.9-81.4)	37.5 (29.6-45.7)	95.1 (92.4-97.4)
		**AD dementia**					
**Number of altered biomarkers**	1 biomarker	74.3 (59.2-85.5)	69.1 (63.7-74.8)	80.0 (50.0-100.0)	68.7 (62.9-74.4)	9.2 (3.5-15.8)	98.9 (97.0-100.0)
	2 biomarkers	76.9 (62.2-88.3)	74.2 (68.9-79.5)	80.0 (50.0-100.0)	73.9 (68.4-79.3)	11.6 (4.6-19.7)	98.9 (97.1-100.0)
	3 biomarkers	81.3 (73.7-87.5)	73.6 (68.2-78.9)	90.5 (76.2-100.0)	72.1 (66.4-77.7)	22.1 (13.9-31.1)	98.9 (97.1-100.0)

**Combinations of biomarkers**	**P-tau 217 + NfL**	65.3 (56.9-73.7)	74.6 (70.5-78.7)	54.1 (37.8-70.0)	76.6 (72.3-80.8)	18.4 (11.5-26.1)	94.5 (91.8-96.8)
	**P-tau 217+ GFAP**	71.2 (63.3-79.5)	78.7 (74.8-82.5)	62.2 (46.7-78.3)	80.3 (76.2-84.2)	23.5 (15.6-32.2)	95.6 (93.3-97.8)
	**GFAP+ NfL**	66.8 (58.8-75.1)	72.9 (68.6-77.2)	59.3 (43.9-75.0)	74.2 (69.8-78.6)	18.3 (11.9-25.8)	94.9 (92.3-97.3)

Supplementary Table 9. Predictive performance measures of blood biomarkers of AD for detecting 10-year all-cause and AD dementia in SNAC-K participants with memory complaints

**Biomarker**	**AUC (95%CI)**	**Correctly classified participants (accuracy) (95%CI)**	**Sensitivity (95%CI)**	**Specificity (95%CI)**	**PPV (95%CI)**	**NPV (95%CI)**
	**All-cause dementia**					
Aβ42/40	66.4 (58.3-73.9)	52.2 (46.5-57.8)	74.5 (62.5-86.0)	47.6 (41.4-54.0)	22.45 (16.5-28.7)	90.2 (84.9-94.9)
P-tau181, pg/mL	81.6 (75.9-87.0)	72.1 (67.1-77.1)	80.4 (68.6-90.9)	70.4 (64.6-75.9)	35.7 (27.1-44.6)	94.6 (91.2-97.8)
P-tau217, pg/mL	80.2 (73.8-86.0)	71.4 (66.5-76.4)	76.5 (64.2-87.8)	70.4 (64.8-75.9)	34.5 (25.9-43.6)	93.6 (89.9-96.9)
T-tau, pg/mL	68.8 (60.5-76.5)	57.1 (51.5-62.8)	76.5 (64.6-88.0)	53.2 (47.1-59.3)	25.0 (18.5-31.9)	91.7 (86.9-96.0)
NfL, pg/mL	80.1 (73.9-85.8)	67.8 (62.5-72.8)	88.2 (78.7-96.3)	63.6 (57.7-69.4)	33.1 (25.3-41.1)	96.4 (93.3-98.8)
GFAP, pg/mL	74.9 (67.3-81.7)	65.1 (59.5-70.4)	78.4 (66.7-89.3)	62.4 (56.2-68.1)	29.9 (22.2-37.8)	93.4 (89.4-96.9)
	**AD-dementia**					
Aβ42/40	61.9 (52.9-70.6)	53.6 (47.9-59.6)	71.4 (53.6-87.5)	51.6 (45.5-57.8)	14.0 (8.6-20.0)	94.2 (90.0-97.8)
P-tau181, pg/mL	73.3 (64.9-81.5)	60.3 (54.6-66.0)	78.6 (62.5-93.3)	58.3 (52.2-64.3)	17.2 (10.9-23.9)	96.1 (93.0-98.8)
P-tau217, pg/mL	75.9 (67.0-84.4)	66.0 (60.6-71.3)	71.4 (54.2-88.0)	65.4 (59.4-71.2)	18.5 (11.4-26.1)	95.4 (92.1-98.3)
T-tau, pg/mL	54.7 (44.1-64.7)	48.9 (43.3-54.6)	67.9 (50.0-84.4)	46.9 (40.7-53.0)	12.3 (7.4-17.6)	93.0 (88.1-97.0)
NfL, pg/mL	69.0 (58.7-78.5)	62.1 (56.4-67.4)	67.9 (50.0-84.6)	61.4 (55.4-67.3)	16.2 (9.6-23.1)	94.6 (90.9-97.6)
GFAP, pg/mL	74.7 (65.6-82.7)	65.6 (59.9-70.9)	82.1 (66.7-95.8)	63.8 (57.8-69.6)	20.0 (12.8-27.4)	97.0 (94.3-99.4)

Supplementary Table 10. Analytical validation and compatibility assessment of Simoa ALZpath p-Tau 217 in SRX

Test	Internal Result SR-X *	Compatibility report with SR-X**	Validation report HD-X***
LOD	0.0003-0.0004 pg/ml	0.0004 pg/mL	0.0003-0.0021 pg/ml
Analytical LLOQ	0.0020-0.0030 pg/ml	0,00433 pg/mL	0.00326 pg/mL
Functional LLOQ	0.0060-0.0090 pg/mL	0,01299 pg/mL	0,00978 pg/mL
Precision profile	Mean Total CV (%)<br>Within Run CV (%): 11<br>Between Run CV (%):13.4<br>Cohort Within 39 Run CV (%): 13.3<br>Cohort Between 39 Run CV (%): 17.6<br>Control1/2 Within 39 Run CV (%): 8.8/6.1<br>Controls1/2 Between 39 Run CV (%): 12/11.2		Mean Total CV (%):5.3<br>Within Run CV (%): 4<br>Between Run CV (%):10
Spike and Recovery	EDTA plasma, peptide spike: 31 % range: 38-43%<br>EDTA plasma, CSF spike: 54%, range 51-57%<br>Serum, peptide spike: 37 % range:31-40%<br>Serum, CSF spike: 49%, range 47-51%		EDTA plasma, peptide spike: 22% range:17-28%<br>EDTA plasma, CSF spike: 50%, range 41-63%
Dilution Linearity	EDTA plasma 3x-24x: 100% (range: 80-120%)<br>Serum 3x-24x: 101% (range 93-108%)		EDTA plasma 3x-24x: 125% (range 101-151%)

Internal Result SR-X*: analytical parameters generated at the Affinity Proteomics Unit, are compared with the reports generated by Quanterix and available online: https://www.quanterix.com/simoa-assay-kits/simoa-alzpath-p-tau-217-assay/

Compatibility report with SR-X: data from the** report published by Quanterix

Validation report HD-X*: data from the** report published by Quanterix .

Supplementary Table 11. Intra and inter assay precision expressed as coefficient of variation (% CV).

	Sample pool			Control 1			Control 2		
	**Conc**	**Within run**	**Between run**	**Conc**	**Within run**	**Between run**	**Conc**	**Within run**	**Between run**
**Aβ-40**	120.4	2.2	5.2	10.1	3.8	6.8	156.1	1.6	3.7
**Aβ-42**	6.6	4.8	7.4	3.4	6.1	8.2	76.8	2.1	4.7
**p-tau 181**	1.5	9.2	13.6	3.6	4.9	7.9	98.0	3.2	5.9
**p-tau 217**	0.14	13.3	17.6	0.8	8.8	12.0	7.7	6.1	11.2
**t-tau**	0.9	10.7	12.3	1.6	5.3	8.5	61.7	2.0	5.2
**NfL**	24.1	7.6	11.6	4.1	11.5	17.6	409.8	4.5	7.4
**GFAP**	194	13.1	21.4	89.8	11.4	17.4	10311	5.8	9.7

Conc: average of the concentrations (pg/mL) obtained in 39 runs.

Within run: CV (%) calculated on the triplicate pool, control 1 and control 2 included in each run (plate). The average CV for all runs is reported.

Between run: CV (%) calculated on the concentration values obtained for pool, control 1 and control 2 across all the runs (plate).
