# Supplementary material for: Blood-based biomarkers of Alzheimer’s disease and incident dementia in the community
Source: Nat Med. 2025 Mar 26;31(6):2027–35. doi: 10.1038/s41591-025-03605-x (PMC12176656; doi:10.1038/s41591-025-03605-x)
Supplement: Supplementary file 2 — Reporting Summary [file 41591_2025_3605_MOESM2_ESM.pdf]

nature portfolio

Corresponding author(s): Giulia Grande

Last updated by author(s): Feb 14, 2025

Reporting Summary

Nature Portfolio wishes to improve the reproducibility of the work that we publish. This form provides structure for consistency and transparency in reporting. For further information on Nature Portfolio policies, see our Editorial Policies and the Editorial Policy Checklist.

Statistics

For all statistical analyses, confirm that the following items are present in the figure legend, table legend, main text, or Methods section.

n/a	Confirmed	
☐	☒	The exact sample size (*n*) for each experimental group/condition, given as a discrete number and unit of measurement
☐	☒	A statement on whether measurements were taken from distinct samples or whether the same sample was measured repeatedly
☐	☒	The statistical test(s) used AND whether they are one- or two-sided<br>*Only common tests should be described solely by name; describe more complex techniques in the Methods section.*
☐	☒	A description of all covariates tested
☒	☐	A description of any assumptions or corrections, such as tests of normality and adjustment for multiple comparisons
☐	☒	A full description of the statistical parameters including central tendency (e.g. means) or other basic estimates (e.g. regression coefficient) AND variation (e.g. standard deviation) or associated estimates of uncertainty (e.g. confidence intervals)
☐	☒	For null hypothesis testing, the test statistic (e.g. *F*, *t*, *r*) with confidence intervals, effect sizes, degrees of freedom and *P* value noted<br>*Give P values as exact values whenever suitable.*
☒	☐	For Bayesian analysis, information on the choice of priors and Markov chain Monte Carlo settings
☒	☐	For hierarchical and complex designs, identification of the appropriate level for tests and full reporting of outcomes
☐	☒	Estimates of effect sizes (e.g. Cohen's *d*, Pearson's *r*), indicating how they were calculated

Our web collection on statistics for biologists contains articles on many of the points above.

Software and code

Policy information about availability of computer code

Data collection	Data were collected through dedicated interviews and related forms (no software used). For biomarker analyses Quanterix SR-X software version: 1.2.0 was used.
Data analysis	All statistical analyses were performed with Stata, version 17 (StataCorp, TX, USA), R version 4.3.1 (The R Foundation for Statistical Computing) and GraphPad Prism 9.

For manuscripts utilizing custom algorithms or software that are central to the research but not yet described in published literature, software must be made available to editors and reviewers. We strongly encourage code deposition in a community repository (e.g. GitHub). See the Nature Portfolio guidelines for submitting code & software for further information.

Data

Policy information about availability of data

All manuscripts must include a data availability statement. This statement should provide the following information, where applicable:

- Accession codes, unique identifiers, or web links for publicly available datasets
- A description of any restrictions on data availability
- For clinical datasets or third party data, please ensure that the statement adheres to our policy

SNAC-K data are sensitive data; thus, they cannot be shared publicly, but raw and analysed de-identified data can be requested by qualified researchers at https://

www.snac-k.se/. The request will be reviewed by SNAC-K Investigators to ensure confidentiality obligations and intellectual property. A data sharing agreement must be signed prior to data release.

Research involving human participants, their data, or biological material

Policy information about studies with human participants or human data. See also policy information about sex, gender (identity/presentation), and sexual orientation and race, ethnicity and racism.

Reporting on sex and gender	Information concerning sex (biological attribute) is available in SNAC-K, whereas gender is not.<br>All analyses have been repeated by sex and reported in supplementary files.
Reporting on race, ethnicity, or other socially relevant groupings	SNAC-K cohort is composed of a sample of white individuals 60+ from an affluent area of Stockholm. No other ethnicities are present in the sample.
Population characteristics	At each study visit, data were collected through standardized procedures, including face-to-face interviews and clinical and laboratory examinations conducted by trained physicians, nurses, and psychologists. Participants were evaluated either at the research center or, for those unable to reach it, at home or at the institution. During the nurse interview, demographic information such as age, sex, and education was obtained. Educational attainment was categorized into elementary school, high school, and college/university or above. Venous blood samples were obtained for DNA extraction, and genotyping was performed to identify Apolipoprotein E (APOE) alleles. Participants were subsequently categorized as either ε4 carriers or ε4-non carriers. To ensure a comprehensive understanding of participants' health status, a thorough clinical procedure was implemented, as detailed elsewhere. This involved medical history collection during physician interviews, clinical (both general and neurological) examinations, diagnostic tests (both instrumental and blood tests), and data from inpatient and outpatient records, medical journals, and the Swedish National Patient Register. Diagnoses were coded according to the International Classification of Diseases, 10th revision (ICD-10), following a clinical review conducted by trained physicians.
Recruitment	The study population consists of adults ≥60 years living in the community or in institutions, from the Kungsholmen district of Stockholm, Sweden. Before the visit, the participant was invited by a letter or a phone call and informed by a detailed letter about the purpose of the study and the duration and interview process. A random sample of 11 age cohorts born between 1892 and 1939 (the youngest and oldest age cohorts were oversampled) was invited to participate in the study. People who agreed to participate were evaluated for the first time between 2001 and 2004. Participants who were <78 years of age were then followed up every six years and participants ≥78 years every three years. The present study is based on data collected at baseline, six years, and 12 years. At baseline, 3363 people were examined (participation rate 73%). Non-participants were older, more likely females, and more likely institutionalized. As a consequence, non-participants may have presented a higher disease burden. A participation rate of 73% remains among the highest reported in population-based studies involving older people.
Ethics oversight	The research protocol for each phase of the SNAC-K study received approval from the Regional Ethical Review Board in Stockholm, and ethical standards of the Declaration of Helsinki were followed throughout the investigation.

Note that full information on the approval of the study protocol must also be provided in the manuscript.

Field-specific reporting

Please select the one below that is the best fit for your research. If you are not sure, read the appropriate sections before making your selection.

☒ Life sciences ☐ Behavioural & social sciences ☐ Ecological, evolutionary & environmental sciences

For a reference copy of the document with all sections, see nature.com/documents/nr-reporting-summary-flat.pdf

Life sciences study design

All studies must disclose on these points even when the disclosure is negative.

Sample size	No sample size calculations have been carried out. The present study is based on the population-based study SNAC-K, which includes at baseline 3363 individuals aged 60+. The sample for this stuy included all 2148 SNACK participants with available serum assays.
Data exclusions	From the initial cohort of SNAC-K participants at baseline (n=3,363), we excluded individuals diagnosed with dementia (n=240) and those with missing information on dementia status (n=10). Of these, 2,555 participants consented to blood sampling, which included a comprehensive health assessment (e.g., hemoglobin, C-reactive protein, and cholesterol levels) and additional samples collected for research purposes. Among the collected blood samples, we excluded participants with missing data in at least one AD blood biomarker (n = 265). Out of the 2,290 dementia-free individuals with available biomarker data, 142 dropped after baseline assessment, leaving a final analytical sample of 2,148 individuals.
Replication	Several sensitivity analyses were performed. For example, we excluded individuals with a MMSE (Mini Mental State Examination) score less than 27, we stratified analyses by age, sex and APOE carriership. We conducted both basic (only age, sex, education) and fully (addinionally adjusted for several comorbidities) adjusted models. Analyes were run multimple times to confirm the findings and data is available for qualified researchers to replicate (see data availability statement).
Randomization	The current study is an obervational study, thus participants were not randomized

Blinding	All serum assays were performed by individuals who were blinded to the clinical data. Clinical diagnoses were performed blided to blood results (e.g. performed before the serum assays were run).

Reporting for specific materials, systems and methods

We require information from authors about some types of materials, experimental systems and methods used in many studies. Here, indicate whether each material, system or method listed is relevant to your study. If you are not sure if a list item applies to your research, read the appropriate section before selecting a response.

Materials & experimental systems

n/a	Involved in the study
☐	☒ Antibodies
☒	☐ Eukaryotic cell lines
☒	☐ Palaeontology and archaeology
☒	☐ Animals and other organisms
☒	☐ Clinical data
☒	☐ Dual use research of concern
☒	☐ Plants

Methods

n/a	Involved in the study
☒	☐ ChIP-seq
☒	☐ Flow cytometry
☒	☐ MRI-based neuroimaging

Antibodies

Antibodies used	The serum concentrations of NfL and GFAP were measured using Simoa Neuro 2-plex B Kit (Quanterix, product number 103520 and Lot# 503409). Simoa Neuro 3-plex A Kit (Quanterix, product number 101995 and Lot# 503659) was used to measure serum Aβ40, Aβ42 and t-tau and Simoa pTau-181 Advantage V2 Kit (Quanterix, product number 103714 and Lot# 503703) was used to measure serum p-tau181. P-tau217 was quantified using the commercial assay Simoa® ALZpath p-tau217 Advantage PLUS developed for the Quanterix HD-X system. For each kit, 25 µL of sample were diluted 1:4 and the assays were performed according to manufacturer instructions. The Quanterix instrument provides, for all proteins, AEB (average enzyme per bead) values for calibrators, controls and samples. The Quanterix SR-X software automatically performs curve-fitting, extrapolation of concentrations and graphical representation using the calibrators, a series of known concentration of an analyte, and a four-parameter logistic (4PL) curve fit.
Validation	https://www.quanterix.com/wp-content/uploads/2022/10/pTau-181-Advantage-V2.1-HD-1-HD-X-Data-Sheet.pdf for p-tau 181<br>https://www.quanterix.com/wp-content/uploads/2020/12/N2PB_SR-X_Data_Sheet_rev01.pdf for NfL and GFAP<br>https://www.quanterix.com/wp-content/uploads/2020/12/Simoa_N3PA_Data_Sheet-SR-X_0.pdf for abeta and total tau<br>Validation of P-tau217 serum assay on Simoa SR-X was carried out as part of the present study and described in the methods and suppementary material

Plants

Seed stocks	NA
Novel plant genotypes	NA
Authentication	NA
